# Mechanism and Structural Insights Into a Novel Esterase, E53, Isolated From *Erythrobacter longus*

**DOI:** 10.3389/fmicb.2021.798194

**Published:** 2022-01-05

**Authors:** Yi Ding, Laiyin Nie, Xiao-Chen Yang, Yang Li, Ying-Yi Huo, Zhengyang Li, Yan Gao, Heng-Lin Cui, Jixi Li, Xue-Wei Xu

**Affiliations:** ^1^Key Laboratory of Marine Ecosystem Dynamics, Second Institute of Oceanography, Ministry of Natural Resources, Hangzhou, China; ^2^School of Oceanography, Shanghai Jiao Tong University, Shanghai, China; ^3^CRELUX GmbH, Munich, Germany; ^4^Teaching Center of Biological Experiments, Zhejiang University, Hangzhou, China; ^5^State Key Laboratory of Genetic Engineering, Shanghai Engineering Research Center of Industrial Microorganisms, School of Life Sciences, Fudan University, Shanghai, China; ^6^School of Food and Biological Engineering, Jiangsu University, Zhenjiang, China

**Keywords:** crystal structure, serine esterase, catalytic pocket, enzyme mutation, pH regulation

## Abstract

Esterases are a class of enzymes that split esters into an acid and an alcohol in a chemical reaction with water, having high potential in pharmaceutical, food and biofuel industrial applications. To advance the understanding of esterases, we have identified and characterized E53, an alkalophilic esterase from a marine bacterium *Erythrobacter longus*. The crystal structures of wild type E53 and three variants were solved successfully using the X-ray diffraction method. Phylogenetic analysis classified E53 as a member of the family IV esterase. The enzyme showed highest activity against *p*-nitrophenyl butyrate substrate at pH 8.5–9.5 and 40^°^C. Based on the structural feature, the catalytic pocket was defined as R1 (catalytic center), R2 (pocket entrance), and R3 (end area of pocket) regions. Nine variants were generated spanning R1–R3 and thorough functional studies were performed. Detailed structural analysis and the results obtained from the mutagenesis study revealed that mutations in the R1 region could regulate the catalytic reaction in both positive and negative directions; expanding the bottleneck in R2 region has improved the enzymatic activity; and R3 region was associated with the determination of the pH pattern of E53. N166A in R3 region showed reduced activity only under alkaline conditions, and structural analysis indicated the role of N166 in stabilizing the loop by forming a hydrogen bond with L193 and G233. In summary, the systematic studies on E53 performed in this work provide structural and functional insights into alkaliphilic esterases and further our knowledge of these enzymes.

## Introduction

Catalyzing the hydrolysis and synthesis of lipids, lipolytic enzymes are essential enzymes not only in the scope of biological processes (Arpigny and Jaeger, 1999; Nardini and Dijkstra, 1999) but also for industrial applications (Stergiou et al., 2013; Ferrer et al., 2016). Esterases can be used as biocatalysts to separate enantiomers in a racemic mixture (Fotheringham et al., 1999; Gonzalo et al., 2001; Monti et al., 2010; Kim et al., 2011; Jose et al., 2016), to catalyze esterification and transesterification reactions (Secundo et al., 2001; Yesiloglu and Kilic, 2004; Aouf et al., 2014), as well as to hydrolyze reactions (Houde et al., 2004). Based on substrate specificity, lipolytic enzymes can be classified into two groups, the lipases (EC 3.1.1.3) that degrade long-chain esters, and the esterases (EC 3.1.1.1) that degrade short-chain esters. According to their conserved sequence motifs and the biological properties, these enzymes could also be classified into 18 families, family I-XVIII (Samoylova et al., 2018).

Family IV esterases, to which E53 belongs, are widely distributed in microorganisms, plants, and animals, specifically degrade short-chain esters (Pereira et al., 2017). The family IV esterase can be characterized by the α/β-fold hydrolase group, and contains two distinct structural domains: an N-terminal CAP domain and a C-terminal catalytic domain (Mandrich et al., 2005; Li et al., 2015). The catalytic reaction relies on the S-H-D/E catalytic triad located in the catalytic domain. The serine initiates the reaction by attacking carbonyl carbon of esters, while histidine and aspartic acid/glutamic acid facilitate the proton transfer from the serine to histidine residue (Li et al., 2014). The family IV esterase contains two conserved motifs, GXSAG and HGGG motif. The HGGG motif (H-G-G-G) is involved in forming the oxyanion hole and plays a vital role in stabilizing the tetrahedral intermediate of the reaction (Oh et al., 2016; Santiago et al., 2018). The GXSAG motif contains the serine, one of the catalytic triad of the enzyme. According to the amino acid residue of X in the GXSAG motif, the family IV esterase can be further classified into GTSAG, GDSAG, and GCSAG subfamily (Jeon et al., 2011; Petrovskaya et al., 2016).

Enzymatic properties of esterases, such as substrate specificity and stress tolerance, are considered as consequences of environmental selection (Castilla et al., 2017; Martinez et al., 2017; Park et al., 2019; Wang et al., 2020). Seawater is weakly alkaline due to the hydrolysis of weakly acidic anions, and high pH is the major characteristics of some marine environments. Esterases obtained from the marine environment often present alkaline adapted features (Huo et al., 2017; Gao et al., 2018). Members of the genus *Erythrobacter* are widely distributed in sea water or sediments environments. Some strains of *Erythrobacter* contain bacteriochlorophyll A and are classified as an essential group of aerobic anoxygenic phototrophic bacteria (AAPB) (Garcia-Chaves et al., 2016; Xu et al., 2018). *Erythrobacter longus* was first discovered in 1982 (Shiba and Simidu, 1982) and to our knowledge, this is the first study focused on the esterase from *E. longus*. The novel esterase, E53, isolated from *E. longus* DSM 6997^T^ in this work, exhibited an alkaline pattern.

In previous studies, key amino acid residues for enzymatic activity and stress tolerance in some esterases have been identified by mutagenesis and structural analysis (Moreth et al., 2007; Kobayashi et al., 2012). Ohara et al. (2014) has conducted elegant work on the pH regulation of an acidic esterase (EstFa) and an alkalophilic esterase (SshEstI): after making a quadruple variant (N248A/P256Q/E257G/S283F of EstFa and A241N/Q249P/G250E/F276S of SshEstI), the pH optimum was reversed for the two esterases. However, relatively limited information is available about the pH adaptation mechanism of the alkaliphilic esterases. In this study, the crystal structure of E53 was determined and the catalytic pocket was structurally and functionally analyzed. Substrate specificity was determined with different carbon chain lengths (C2–C16) *p*-nitrophenyl (NP) esters and specific activities were observed for C4, C6, and C8 substrates. The optimal reaction conditions were pH 8.5–9.5 at 40°C. Structurally, the catalytic pocket of E53 could be defined as three regions: the catalytic core region R1, the pocket entrance channel R2 and the pocket end area R3. To investigate the role of each region and identify the crucial amino acid residues, we did structural alignment to other esterases as well as conducted mutagenesis studies. Mutations in the R1 region up or down regulated the enzyme activity drastically, demonstrating that the enzyme activity is sensitive to changes in the R1 region. From the structure, a “bottleneck” could be identified in the R2 region, and by broadening this bottleneck, the enzyme activity was successfully improved. N166A variant in the R3 region had a very interesting effect on the pH pattern: while the activity at neutral pH remained the same as the wild type (WT) E53, the activity at pH 9.0 decreased nearly half. Taken together, our study has identified vital amino acids for determining pH patterns and enzymatic activity of E53, providing a basis for further exploration of its potential biotechnological application.

## Results

### E53 Is an Alkalophilic Esterase Belonging to the Microbial Family IV

**FIGURE 1:**
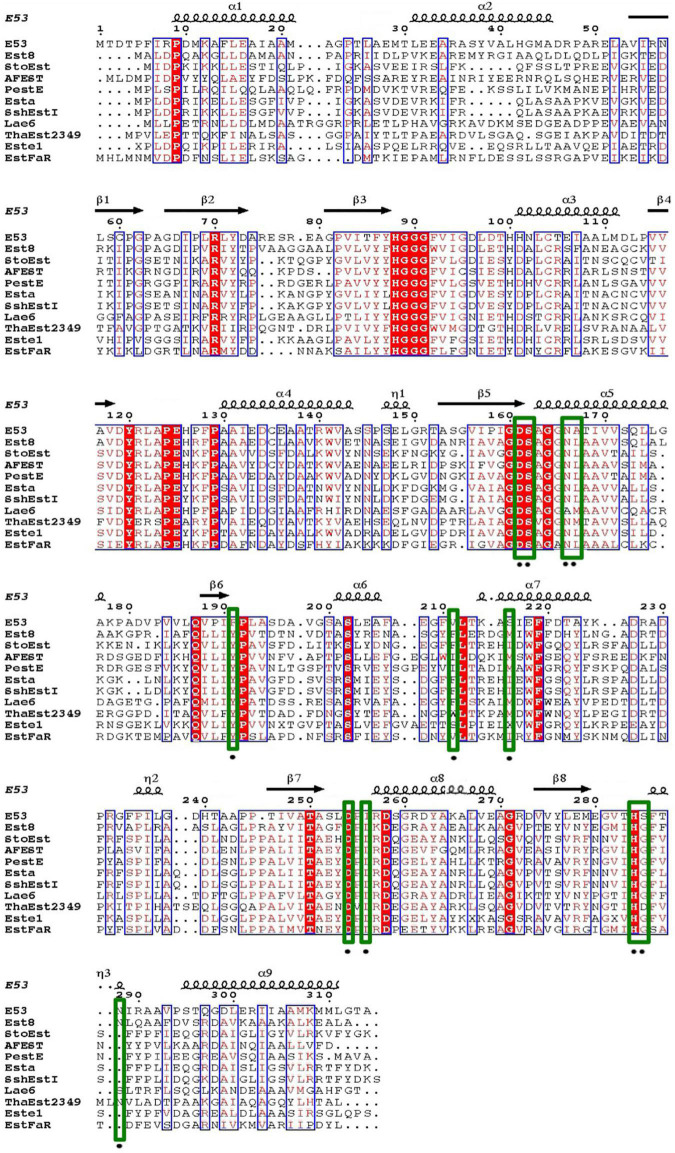
Sequence alignment of E53 and its homologous proteins. Amino acids conserved in all sequences used for alignment were labeled with a red-colored background. Comparative conserved amino acids were labeled with red color. The mutated sites in this study were labeled with green-colored frames.

An alkalophilic esterase, E53, was identified and characterized from a marine bacterium *E. longus* DSM 6997^T^. The amino acid sequence of E53 was aligned with known esterases available in GenBank and Protein Data Bank (PDB) database (Supplementary Table 1). The amino acid sequence of E53 contained a conserved HGGG motif and a typical catalytic motif (GXSAG), indicating its close relationship with microbial family IV esterases (Figure 1). E53 and *Erythrobacter* sp. esterase EAQ30367 (UniProt ID: A3WBQ5) share a sequence identity of 81.1%. The classification of E53 as a new microbial family IV esterase was verified by phylogenetic analysis (Supplementary Figure 1).

**FIGURE 2:**
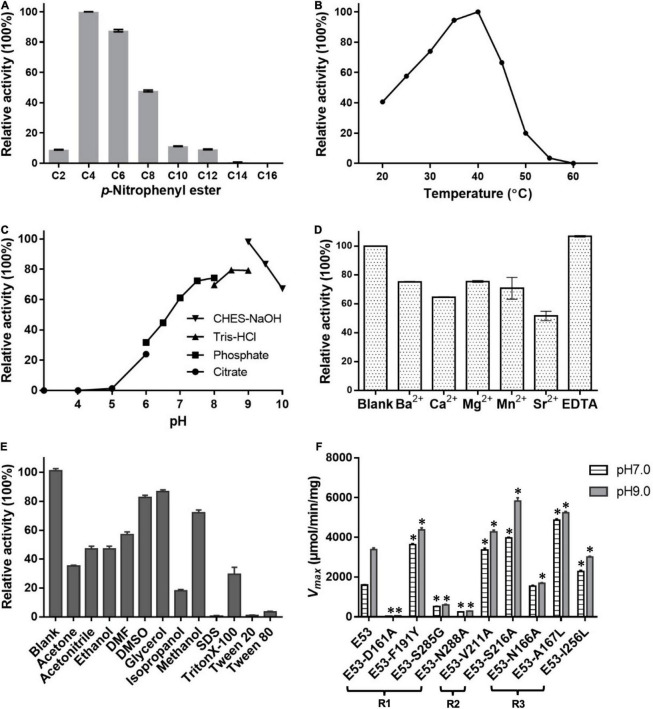
Enzymatic features of E53 and the variants. **(A)** Substrate specificities of E53. The catalytic activity was measured under pH 9.0 and 40°C. The catalytic activity with the C4 substrate was defined as 100% activity. **(B)** Effect of temperature on the enzyme activity: the optimum temperature was 40°C. The C4 substrate was used for measurements **(B–F)**. **(C)** Effect of pH value on the enzyme activity: optimum pH was in the range of 8.5–9.5. **(D)** Tolerance of E53 on EDTA and different divalent cations. The reaction without ions was used as a control. **(E)** Effects of organic solvents on the enzymatic activity of E53. The reaction without any organic solvent was used as a control. **(F)** Maximum velocity (*V*_*max*_) of E53 and its variants. The catalytic assay was under pH 7.0 and pH 9.0 conditions, 40°C. The data are shown as mean ± SD (*n* = 3). * Represents a significant difference from the WT E53 in the same conditions (*t*-test, p < 0.05). For some points, the error bars would be shorter than the height of the symbol.

Different carbon chain lengths (C2–C16) *p*-NP esters were tested and E53 specifically catalyzed the hydrolysis of C4, C6, and C8 substrates, demonstrating its function as an esterase (Figure 2A). The optimum temperature of E53 activity was 40°C and the catalytic capability attenuated when the temperature was higher than 45°C (Figure 2B). The catalytic activity was observed at a wide range of pH 5.0–10.0 (Figure 2C), and the optimum pH was in the range of 8.5–9.5. The catalytic activity of E53 in NaCl gradient was measured under pH 9.0 and 40°C. E53 kept 91.0% and 86.8% of enzyme activity in 1 M and 2 M NaCl, respectively. The activity reduced to 59.5% in 3 M NaCl and 15.4% in 4 M NaCl, and was not detectable in 5 M NaCl (Supplementary Figure 2). We have also explored the impact of divalent cations and organic solvents on the activity of E53. Results suggested that E53 exhibited good tolerance on several metal ions: activity was retained to more than 50% in all tested cations (Ba^2+^, Ca^2+^, Mg^2+^, Mn^2+^, Sr^2+^) and EDTA did not show any inhibition to the enzymatic activity (Figure 2D). In organic solvents like DMSO, DMF, glycerol, acetone and acetonitrile, E53 presented at least 35% activity. Methanol, ethanol and isopropanol were tested as alcohol, and the activity level varied from 70% (in methanol) to 18% (in isopropanol). The relative activity in methanol, ethanol and isopropanol showed the same trend as the polarity of the solvents. We have also measured the activity in the presence of detergents, including SDS, TritonX-100, Tween 20 and Tween 80. As a mild detergent, about 30% activity was detected in TritonX-100, while in the other three detergents the enzyme was not functional (Figure 2E).

### Structural Comparison of E53 and Other Esterases

**FIGURE 3:**
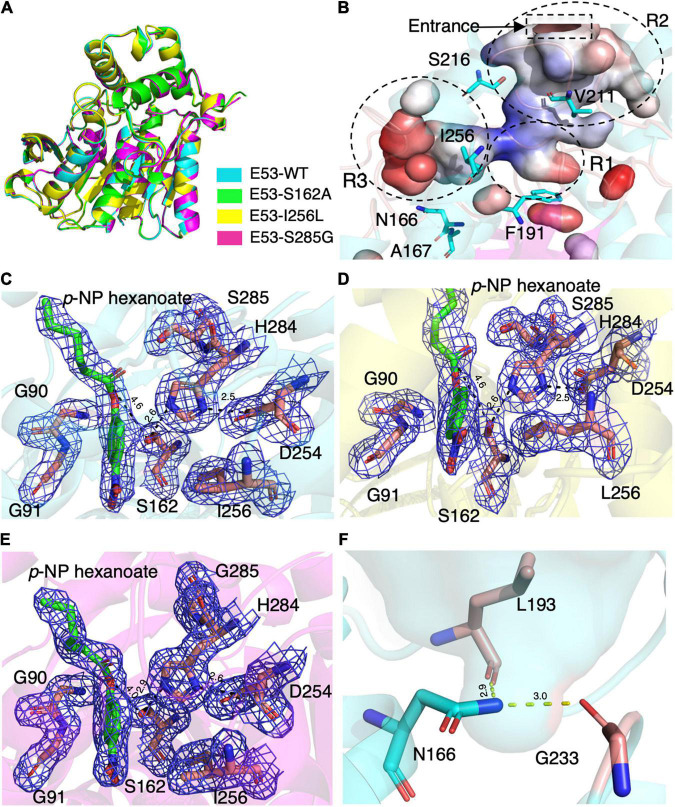
The crystal structures of E53 and its variants. **(A)** The superimposed global structure of E53 (cyan), S162A (green), I256L (yellow), and S285G (magenta). **(B)** The catalytic pocket of E53. The catalytic pocket was divided into R1, R2, and R3 regions. The mutated residues were shown as stick models. Electrostatic surface is shown: red color represented negative potential and blue color represented positive potential. **(C)** The C6 substrate and the catalytic triad of WT E53. **(D)** The C6 substrate and the catalytic triad of I256L. **(E)** The C6 substrate and the catalytic triad of S285G. All the electron density maps were contoured to 1.0 σ at the 2Fo-Fc map. **(F)** The N166 and its interaction with L193 and G233.

**FIGURE 4:**
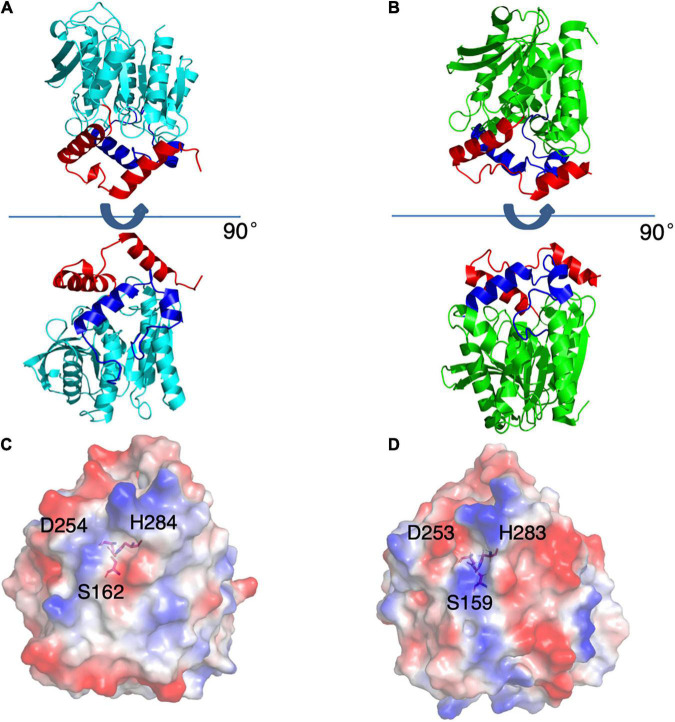
Structures of E53 and Est8. **(A)** The 3D structure of E53, including the α/β-hydrolase fold (cyan), the carboxyl edge of the β-sheet (blue), and the cap subdomain (red) **(B)** The 3D structure of Est8 (PDB: 4YPV), including the α/β-hydrolase fold (green), the carboxyl edge of the β-sheet (blue), and the cap subdomain (red). **(C,D)** The surface contact potential of E53 and Est8, respectively. The catalytic triad was shown as stick models. Blue represents positive potential, and the red represents negative potential.

To understand the mechanism of this novel alkalophilic esterase, we have determined the structures of the WT protein and three variants: S162A, I256L, and S285G to 1.70–2.09 Å, all in the presence of the C6 substrate (Table 1). The 3D structure of the enzyme was determined by molecular replacement using the esterase Est8 (PDB: 4YPV) as a search model. The overall E53 structure presents as a monomer, in agreement with the oligomeric state in solution (Supplementary Figures 3, 4). The WT E53 with 314 amino acids has a typical α/β-hydrolase fold. It contains eight β-strand and nine α-helices (Figure 1). Two α-helices (α1 and α2) at the N-terminal region form a CAP domain, the conserved domain present in all microbial family IV esterases (Figures 3A, 4A). The CAP domain covers the catalytic site, providing structural integrity to the protein as well as playing the role of the substrate entrance channel. The β-strands conform parallel β-sheets in the order of β1, β2, β3, β4, β5, β6, β7, and β8, with four α-helices (α1, α2, α3, and α9) on the one side and five α-helices (α4, α5, α6, α7, and α8) on the other side. The oxyanion hole was composed of G90 and G91 within the conserved HGGG motif (amino acids No. 88-91) (Figure 3C). The catalytic triad was composed of S162, D254, and H284. We have prepared S162A, D254A and H284A variants (Supplementary Figure 5) and mutating the three residues to alanine (S162A, D254A, H284A) resulted in almost total loss of activity.

**TABLE 1:** Data collection of E53 and its variants.

Item	E53-WT	E53-S162A	E53-I256L	E53-S285G
PDB No.	7W8N	7CI0	6KF5	7CIH
**Data collection**				
Resolution range (Å)	48.71–1.752 (1.815–1.752)	47.35–1.702 (1.763–1.702)	37.33–2.09 (2.17–2.09)	48.54–1.79 (1.85–1.79)
Space group	P 2_1_ 2 2_1_	P 2_1_ 2 2_1_	P 2_1_ 2 2_1_	P 2_1_ 2 2_1_
Cell dimensions (Å,°)	a = 70.58, b = 129.84 c = 221.08, α = β = γ = 90	a = 70.40, b = 129.77, c = 220.66, α = β = γ = 90	a = 70.77, b = 129.60, c = 219.70, α = β = γ = 90	a = 70.66 b = 129.69 c = 219.57, α = β = γ = 90
Unique reflections	203632 (20064)	221017 (21745)	119277 (11664)	189466 (18072)
R-merge	0.02979 (0.3273)	0.01532 (0.2582)	0.171 (1.043)	0.02048 (0.3826)
Mean I/σ (I)	14.20 (2.13)	20.16 (2.49)	17.40 (2.43)	18.53 (2.05)
Completeness (%)	99.86 (99.44)	99.79 (98.94)	99.56 (98.83)	99.47 (95.93)
Wilson B-factor	26.03	27.09	27.20	30.11
R-pim	0.02979 (0.3273)	0.01532 (0.2582)	0.049 (0.298)	0.02048 (0.3826)
R-meas	0.04213 (0.4629)	0.02166 (0.3651)	0.178 (1.086)	0.02896 (0.541)
CC 1/2	0.998 (0.85)	1 (0.918)	1 (0.893)	0.999 (0.825)
**Refinement statistics**				
Reflection used in refinement	203632 (20064)	220838 (21718)	119096 (11664)	189423 (18072)
R-work^c^	0.1641 (0.2821)	0.1711 (0.2733)	0.1829 (0.2258)	0.1690 (0.3035)
R-free^d^	0.1786 (0.2932)	0.1908 (0.2919)	0.2139 (0.2595)	0.1901 (0.3254)
Total No. atoms	11006	10881	10291	10685
No. atoms (protein)	9136	9128	9111	9196
No. atoms (ligands)	439	392	138	238
No. water molecules	1431	1361	1042	1251
**RMSD**				
RMS bonds (Å)	0.011	0.010	0.08	0.012
RMS angles (°)	1.14	0.99	1.21	1.41
Ramachandran favored (%)	97.64	97.07	96.73	96.83
Ramachandran allowed (%)	2.36	2.93	3.27	3.17
Ramachandran outliers (%)	0.00	0.00	0.00	0.00
Clashscore	9.54	9.21	4.63	6.59
MolProbity score	1.60	1.80	1.44	1.56
Average B-factor	32.13	33.54	28.80	35.88

*^a^Statistics for the highest-resolution shell are shown in parentheses.*

*^b^R_merge_ = Σ | Ii - < I > | /Σ | I|, where I_i_ is the intensity of an individual reflection and is the average intensity of that reflection.*

*^c^R_work_ = Σ | | Fo| —| Fc| | /Σ | Fo|, where F_o_ and F_c_ are the observed and calculated structure factors for reflections, respectively.*

*^d^R_free_ was calculated as R_work_ using the 5% of reflections that were selected randomly and omitted from refinement.*

Though the crystallization of all four proteins was carried out in the presence of the C6 substrate, the electron density of the substrate molecule was only observed in the crystal structures of E53, I256L, and S285G (Figures 3C–E), but not in the S162A variant. The superimposed structures did not suggest significant differences in global structure and catalytic center among WT E53 and the three variants (Figure 3A). The distances among the catalytic triad in WT and mutated E53 were 2.6–2.9 Å for H284Nε2 to S162Oγ and 2.5–2.6Å for H284Nδ1 to D254Oδ2 (Figures 3C–E).

The catalytic pocket of E53 was formed by seven α-helices (α1–α7) and four β-sheets (β5–β8). The catalytic pocket presented a mirrored “L” structure, and the catalytic center was located in the node of it (Figure 3B). In order to describe the catalytic pocket in a concise way, three regions were defined: R1—catalytic center region, R2—pocket entrance channel area and R3—end area of pocket. The R1 region is encircled by two α-helices (α3 and α7) and four β-sheets (β5–β8) containing the catalytic center. The R2 region is the space between the CAP domain and the catalytic domain. It is the entrance of the catalytic pocket, giving access to substrates. The R3 region defined by four α-helices (α4–α8) is an extensive area of the catalytic pocket (Figure 3B and Supplementary Figure 6).

The C6 substrate was modeled based on the electron density at the catalytic center of the pocket between the oxyanion hole (G90 and G91) and two residues of the catalytic triad (S162 and H284). The *p*-NP substrate was accommodated in the R3 region while the carboxyl acid was pointing in the direction of the pocket entrance. The catalytic reaction is achieved by the nucleophilic attack from serine and histidine-mediated proton transfer. The distance between S162Oγ to the carbonyl carbon of ester bond was 4.6 Å in WT E53, 4.6 Å in E53-I256L, and 4.0 Å in E53-S285G. Such distance could allow the catalytic process to happen (Figures 3C–E).

### Structural Comparison of E53 and Other Esterases

To compare the catalytic pocket of E53 with other enzymes from the same family, the structures of microbial family IV esterases were collected from the ESTHER and PDB databases (Supplementary Table 2). The crystal structures of 38 WT esterases, including 16 bacterial esterases, 7 archaeal esterases, 3 fungi-originated esterases, and 12 metagenomic-originated esterases, were analyzed. The structural comparison of E53 and other esterases exhibited significant structural diversity in the catalytic pocket. Only one third of these esterases (6 from archaea, 3 from bacteria and 2 from fungi) had a similar R3 region (Supplementary Table 2 and Supplementary Figure 7). Interestingly, these 12 esterases (including E53) have been reported to be alkalophilic or acidophilic and were mostly isolated from extreme pH or temperature environments (Manco et al., 2000; Martinez et al., 2013; Ohara et al., 2014). Among the 12 esterases, the archaeal enzymes are all thermophilic: the optimal temperature is between 80 and 90^°^C (Palm et al., 2011; Angkawidjaja et al., 2012). The pH pattern is more diverse: Sto-Est from *Sulfolobus tokodaii*, SshEstI from *Sulfolobus shibatae* DSM5389 and PestE from *Pyrobaculum calidifontis* have their pH optimum in the range of 7.0–9.0 (Hotta et al., 2002; Palm et al., 2011; Angkawidjaja et al., 2012; Ohara et al., 2014) while EstFa_R from *Ferroplasma acidiphilum* is a slightly acidophilic carboxylesterase and showed the highest activity at pH 5.0 (Ohara et al., 2014). The two fungal esterases share similar optimal conditions: RmEstA from *Rhizomucor miehei* catalyzes the reaction best at 50°C and pH 7.5 while TmelEST2 from *Tuber melanosporum* has its maximum activity at 68.3°C and pH 7.0 (Yang et al., 2015; Cavazzini et al., 2017).

### Mutagenesis Study of E53 Disclosed the Function of R1, R2, and R3 Regions

To elucidate the function of the R1, R2, and R3 regions, we have selected 9 residues covering all three regions and did thorough functional studies.

Four amino acids in the R1 region: D161, F191, S285, and N288, were mutated to varying residues. Amino acids at positions 191 and 285 were conserved in microbial family IV esterases but not in E53 (Figure 1). The conserved tyrosine was replaced by phenylalanine at position 191, while the conserved glycine was replaced by serine at position 285 in E53 (Figure 1). D161 is a conserved residue and part of the “GDSAG” motif, while N288 shows much less conservation; both are located adjacent to the catalytic center hence have been chosen for mutagenesis study. In our work, F191 and S285 in E53 were mutated to the conserved amino acids, and D161 and N288 were replaced with alanine. The variants, D161A, S285G, and N288A, did not change the specificity but dramatically reduced the enzymatic activity (> 90% loss of function) on *p*-NP ester hydrolysis (Figure 2F), demonstrating their crucial roles in the catalytic reaction. Interestingly, the F191Y variant improved the enzymatic activity of E53 by around 30% and the *Km* value decreased, suggesting a higher substrate affinity (Figure 2F and Supplementary Table 3). This result showed that compared with the conserved residue tyrosine, phenylalanine exhibited adverse effects on enzymatic activity in E53.

In the R2 region, the crystal structure of the WT E53 exhibits a structural bottleneck close to the entrance of the pocket, determined by V211 and S216 (Figure 3B). With the intention to expand the size of this channel, we made V211A and S216A variants. As a result, V211A and S216A exhibited higher relative activity comparing to the WT enzyme. In terms of enzyme kinetics, a significant increase in *V*_*max*_ and *Km* value under both neutral and alkaline conditions was achieved (Figure 2F and Supplementary Table 3). The higher *Km* values suggested lower substrate affinity, which is particularly notable. This, however, makes sense in this case, as the enlarged pocket would allow the substrate binding in a looser manner. This clearly proved that appropriate expansion of the entrance could enhance the accessibility of substrates and thus facilitate the catalytic capability of the enzyme.

The R3 region is an extended area of the catalytic pocket in E53 (Figure 3B). The crystal structure of WT and mutated E53 with the C6 substrate exhibited that the R3 region was not directly involved in the substrate binding or catalytic reaction (Figure 3B). To explore its role, three variants, N166A, A167L, and I256L were prepared for the enzymatic analysis under neutral and alkaline conditions (Figure 2F). A167 and I256 were conserved in microbial family IV esterases but not in E53 (Figure 1). The mutation of alanine to conserved leucine at position 167 resulted in 200–400% higher activity and lower *Km* than the WT protein under neutral and alkaline conditions (Figure 2F and Supplementary Table 3), indicating that the residue had a great impact on the catalytic activity. On the contrary to A167L, mutating the I256 to the conserved leucine of this family did not change the activity nor the *Km* significantly. So far, all the investigated variants showed the same trend of impact in the enzyme activity under neutral and alkaline conditions. Interestingly, N166A was an exception: it did not affect the catalytic activity under the neutral condition (< 10% discrepancy on activity), but inhibited 50% activity in the alkaline condition; the *Km* was also more severely affected under the alkaline condition (Supplementary Table 3). We analyzed the effect of pH values on the N166A variant (Supplementary Figure 8). The result disclosed that the N166A variant was less sensitive to pH change: unlike the WT protein, there is nearly a “plateau” from pH 7.5 to 9.0. The optimum pH was shifted to 7.5, however much less pronounced than the WT protein (Figure 2C and Supplementary Figure 8). In addition, at pH 6.0, the WT protein retained about 25% of its maximum activity while N166A had only 5%, but from pH 6.5 to 7.0, N166A showed about 20% higher relative activity than the WT protein, clearly indicating this variant is relatively more active under neutral conditions. The loss of the amide on the side chain of N166 might undermine its interaction with the carbonyl oxygen of L193 and G233 (Figure 3F), thus weakening the molecular stability under alkaline conditions. This is a strong indication that N166 was one of the critical amino acids for pH regulation (Figure 2F).

## Discussion

### The R1 Region Had Significant Impacts on Catalytic Activity, Revealed by Mutagenesis Study and Detailed Structural Comparison Between E53 and Est8

**FIGURE 5:**
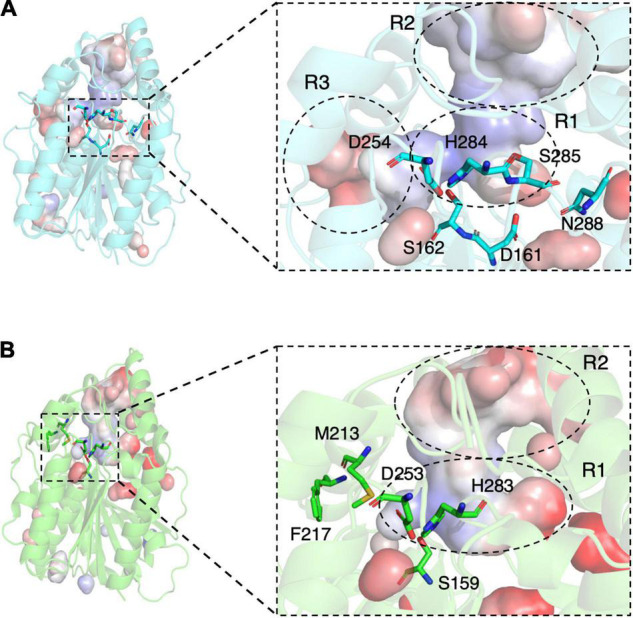
The catalytic pockets of E53 and Est8. **(A)** The catalytic pocket of E53. The catalytic triad, S162, D254 and H284 were located in the R1 region. The D161, S285 and N288 were targets of mutagenesis in the R1 region. **(B)** The catalytic pocket of Est8. S159, D253, and H283 were the components of the catalytic triad. The M213 and F217 were mutated in the previous study (Pereira et al., 2017).

As the esterase Est8 from metagenome exhibited the highest amino acid sequence identity (43%) with E53 (Supplementary Table 1), and shared similar enzymatic features, such as pH optimum (7.0–9.0) and temperature optimum (25–45°C) (Pereira et al., 2017), we did structural and functional analysis of these two proteins in more details. The overall structural overview of the two proteins is shown in Figures 4A,B. The main differences between the two enzymes were the structure of the catalytic pocket and substrate specificity. Compared with E53, there is much less space among four α-helices (α4 - α8) in Est8. Moreover, the catalytic pocket of Est8 lacked the R3 region in E53 (Figures 5A,B). The catalytic pocket of E53 is composed of acidic, basic and neutral amino acids, and the catalytic triad is mainly wrapped by neutral amino acids, whereas the catalytic pocket of Est8 is mainly composed of basic and acidic amino acids, and the catalytic triad is in the alkaline pocket (Figures 4C,D, 5A,B). The Est8 specifically catalyzed the hydrolysis of C2 substrate (Pereira et al., 2017), and E53 catalyzed the hydrolysis of C4, C6, and C8 substrates (Figure 2A). The mutations of Est8, which aimed at expanding the size of the pocket (M213A and F217A, both located close to the catalytic pocket of Est8), did not change the substrate specificity significantly but dramatically reduced the enzymatic activity (90% loss of function) (Pereira et al., 2017). Similar to the mutagenesis analysis in Est8, the mutation of E53-D161A, E53-S285G, and E53-N288A at the R1- catalytic center region significantly reduced catalytic activity, making the evaluation of substrate specificity impossible.

Despite the fact that S285 is adjacent to H284, a residue of the catalytic triad, the crystal structure of E53-S285G did not exhibit obvious relocation of the catalytic triad (2.7Å for H284Nε2 to S162Oγ, and 2.6Å for H284Nδ1 to D254Oδ2) compared with WT E53 (2.9 Å for H284Nε2 to S162Oγ, and 2.6Å for H284Nδ1 to D254Oδ2) (Figures 3C,E). D161, S285, and N288 are all located close to the catalytic center, and functional characterization of these variants disclosed loss of enzymatic activity. As part of the “GDSAG” motif, mutating D161 to A resulted in loss of function was not surprising. In the case of N288A, replacing asparagine with alanine could cause disruption to the local interaction network, impairing the activity dramatically. The unexpected outcome was S285G: most esterases have glycine at position 285; after mutating the serine to the more conserved glycine, only 20–30% of activity was detected. From the structure, the shortest distance from S285 to the bound substrate is 3.0 Å, suggesting S285 could be involved in the coordination of the substrate. Substituting this serine with glycine could impede this interaction, leading to the decrease of the activity. On the contrary, the replacement of phenylalanine with tyrosine at position 191 improved the catalytic activity under both neutral and alkaline conditions, indicating that hydroxyl on tyrosine residue might be involved in the catalytic activity of the enzyme. The amino acid replacement did not change the size of the residue dramatically but introduced an additional hydrogen bond that improved the structural stability of the pocket area. It is indicated that the non-covalent bonds at the R1 region and stability of the catalytic center played key roles in the catalytic activity of the enzyme.

### The R3 Region Plays an Important Role in the pH Pattern of E53

Few studies concerning the pH pattern of esterases have been reported. The only related study is from an archaeal esterase, an alkalophilic esterase SshEst (Ohara et al., 2014). Similar to E53, the catalytic pocket of SshEst contains the R3-end region of pocket. The secondary structures, α5 and α8 helices, were involved in the constitution of the R3-end region of pocket in both SshEst and E53 (Supplementary Figure 9). The replacement of the amino acids at α8 helix of SshEst (Q249 and G250) changed the optimum pH from 8.0 to 6.5 (Ohara et al., 2014).

In our study, three residues in the R3 region were selected for structural and functional studies. A167L achieved higher activity under both neutral and alkaline conditions. In the case of I256L, the structure of WT E53 had hardly changed with that of E53-I256L (Supplementary Figure 10). The distance between the S162Oγ and the carbonyl carbon of the ester bond in the *p*-NP substrate was 4.6 Å in both WT E53 and I256L. The comparative distances among the catalytic triad were 2.6 Å between S162Oγ to H284Nε2 and 2.5 Å between H284Nε2 to D254Oδ2 in both of them. As shown in the structures, the I256L mutation did not have a significant effect on the protein structure, especially at the catalytic area. While E53-I256 was an exception in the sequence compared with other microbial family IV esterases, it did not lead to the significant discrepancy in either protein structure or activity of hydrolysis of C6 substrate. However, the single point mutation (E53-N166A) in the R3 region changed the pH pattern of E53. The N166 is located at the α5 helix. It is neighbored to the SDSAG motif, and is conserved in microbial family IV esterases with few exceptions (Figure 1). Its mutation dramatically impaired the enzymatic activity under alkaline conditions and an increased *K*_*m*_ value was observed under neutral and alkaline conditions (Supplementary Table 3). The hydrogen bond established *via* the side chain from asparagine with the amino acids L193 (2.9 Å between the amide and carbonyl oxygen) and G233 (3.0 Å between the amide and carbonyl oxygen) (Figure 3F) may contribute to the stability of the enzyme under the alkaline condition. It is indicated that both L193 and G233 were located at the loops that constituted the R3 region (the loop between β6 and α6 for L193, and the loop between α7 and β7 for G233). The amide at the side chain of the N166 contributed to fixing the two loops with α5 helix, and thus, stabilizing the R3 region of E53 (Figure 3F). The loss of the side chain of the N166 will impair the molecular interactions among the structural elements that were involved in the R3 region constitution and thus undermine catalytic activity in alkaline conditions.

In conclusion, the R3-end region of the catalytic pocket might play an essential role in pH pattern determination in a group of microbial family IV esterases, while more studies focusing on family IV esterases are needed to get a solid conclusion.

## Materials and Methods

### Bacterial Cultivation and Recombinant Plasmid Construction

*Erythrobacter longus* CGMCC 1.8459^T^ (DSM 6997) was obtained from the China General Microbiological Culture Collection (CGMCC). The genome sequence accession number of *E. longus* DSM 6997^T^ is JMIW01000001 and the *e53* gene is located in the 562979–563923 region^1^. The amino acid sequence accession code of E53 is WP_034957919. Cells were cultivated in marine 2216 medium (Difco, pH 7.5) at 30°C. *Escherichia coli* BL21 (DE3) was cultivated in LB medium (pH 7.0) at 37°C. Genomic DNA of *E. longus* was extracted with a bacterial genomic DNA purification kit (Thermo Fisher Scientific, United States). The WT *e53* gene was amplified by polymerase chain reaction (PCR) with the primers listed in the supporting information (Supplementary Table 4). The pSMT3 SUMO-tag system was used in this study. The SUMO-tag contains six histidines at the N-terminal region, which can bind with the Ni-NTA affinity column. The PCR product and backbone of pSMT3 plasmid were digested by *Bam*HI and *Xho*I (New England Biolabs, United States) at 37°C for 1 h, and followed by kit-based DNA purification (Thermo Fisher Scientific, United States) and T4 DNA ligase-mediated ligation (New England Biolabs, United States). The single amino acid mutation was introduced *via* the kit-based mutation system (Fast Mutagenesis System, TransGen Biotech) with the WT gene as a template. *E. coli* BL21 (DE3) competent cells were transformed with the constructed plasmid by heat-shock approach. The recombinant *E. coli* BL21 (DE3) clones were selected on LB plates with kanamycin (50 μg/ml). The inserts were confirmed by PCR and DNA sequencing.

### Protein Expression and Purification

The *E. coli* BL21 (DE3) cells were cultivated at 37°C in LB medium supplemented with kanamycin (50 μg/ml). The expression of WT E53 and its variants was induced by the addition of 0.5 mM isopropyl-β-D-thiogalactopyranoside (IPTG) for 16 h at 20°C (Angkawidjaja et al., 2012). Cells were harvested and disrupted by an ultrasonic homogenizer (Scientz-IID, China). The lysates were subsequently purified by Ni-NTA affinity chromatography (Thermo Fisher Scientific, United States) using imidazole step elution. The washing buffer contained 20, 50, 100, 250, and 500 mM of imidazole and the target protein was eluted at 250 mM imidazole. The pure E53 protein with SUMO-tag was eluted with NTA-250 buffer containing 20 mM Tris-HCl, pH 8.0, 100 mM NaCl, 250 mM imidazole. The elution was incubated with Ulp1 protease (Thermo Fisher Scientific, United States) at a ratio of 1 μg of Ulp1 per 2 mg protein at 16°C for 1 h and the SUMO-tag was removed by a reverse Ni-NTA affinity chromatography (Huo et al., 2017). The SUMO-tag-free protein with an anticipated molecular weight of 33.13 kDa and PI of 4.73 (calculated by ExPASy (Gasteiger et al., 2003) based on amino acid sequence) was injected onto a Superdex 200 16/600 column. The column was equilibrated in 20 mM Tris-HCl, 100 mM NaCl, pH 8.0 prior to the protein sample run. The purified protein was verified *via* sodium dodecyl sulfate-polyacrylamide gel electrophoresis (SDS-PAGE) using 12% polyacrylamide gels. The Superdex 200 16/600 GL (GE, United States) column was calibrated with protein size markers, thyroglobulin (670 kDa), gamma globulin (158 kDa), ovalbumin (44 kDa), myoglobin (17 kDa), and vitamin B12 (1.35 kDa). The molecular mass (Mw) of the WT E53 was experimentally determined based on *K*_*av*_. *K*_*av*_ = (*Ve* − *Vo)*/(*Vc* − *Vo*) where *Ve* = elution volume, *Vo* = column void volume, *Vc* = geometric column volume. The protein concentration was determined by the Bradford method.

### Biochemical Characterization of E53 and Its Variants

The substrates *p*-nitrophenyl (NP) acetate (C2), *p*-NP butyrate (C4), *p*-NP caprylate (C8), *p*-NP decanoate (C10), *p*-NP laurate (C12), *p*-NP myristate (C14), and *p*-NP palmitate (C16) were purchased from Sigma-Aldrich (United States) and *p*-NP hexanoate (C6) was purchased from TCI (Japan). The standard reaction was carried out with 0.02–0.10 μg of purified E53 or its variants (due to the difference of the activity among WT enzyme and variants) in a 1.0 ml reaction mixture containing 100 mM CHES-NaOH (pH 9.0) buffer and 1 mM *p*-NP butyrate esters. The enzyme activity was determined at 40°C at 405 nm using DU800 UV/Visible spectrophotometer (Beckman, United States). The absorbance values were measured every 15 s for 2 min. The obtained rate of change is used for subsequent enzyme activity determination. All experiments were performed in triplicate and corrected for substrate autohydrolysis. The kinetic parameters were obtained using *p-*NP butyrate as a substrate at different concentrations ranging from 0.05 to 4.00 mM under 40°C and Tris-HCl buffer (pH 7.0 and pH 9.0). The kinetic parameters were calculated by analyzing the slopes of the Michaelis-Menten equation using GraphPad Software (GraphPad Inc., United States). The statistical analysis of *t-*test was calculated by Quickcalcs of GraphPad Software^2^. The optimum pH for enzyme activity was carried out with 100 mM citrate buffer (pH 3.0–6.0), potassium phosphate buffer (pH 6.0–8.0), Tris-HCl buffer (pH 8.0–9.0) and CHES - NaOH buffer (pH 9.0–10), respectively. The reactions at different pH values were measured at 348 nm, the pH-independent isosbestic wavelength of *p*-NP butyrate. The optimum temperature for enzyme activity was measured over a range of 15–60°C with an interval of 5°C. The NaCl pattern was measured using *p*-NP butyrate as a substrate at optimum condition (CHES-NaOH 9.0, 40°C) with NaCl concentrations ranging from 0 to 5.0 M. The effects of various metal ions and the chelating agent ethylenediaminetetraacetic acid (EDTA) were examined at a final concentration of 10 mM. The effects of various organic solvents were examined at a final concentration of 5% (v/v). The product was measured after 5 min of catalytic reaction in the presence of NaCl, organic reagent, or ions under the optimal buffer conditions.

### Crystallization and Structure Determination

The crystal of E53 protein was obtained using a hanging drop method by mixing 1.0 μL of 20 mg/ml protein with 1.0 μL of reservoir solution at 20°C. The reservoir buffer contained 0.05 M CaCl_2_, 0.1 M Bis-Tris, pH 6.5, and 25% (v/v) PEG MME 550. The crystals appeared overnight and grew to their maximum sizes after 5–7 days. The crystals were soaked with 10 μM of C6 substrate acetonitrile solution for 30 s before harvesting. The crystal samples were tested in BL17U1 and BL19U1 beamlines at the National Center for Protein Sciences Shanghai and Shanghai Synchrotron Radiation Facility, China. The X-ray diffraction datasets were integrated, scaled and merged by the HKL3000 program (Minor et al., 2006). Phases were obtained by molecular replacement using Phaser (McCoy et al., 2007) with the esterase Est8 PDB coordinates (PDB no. 4YPV) as the initial model. The crystallographic structure refinement was carried out by Refmac5 (Murshudov et al., 2011; Kovalevskiy et al., 2018) in the CCP4 software suite (Winn et al., 2011) and Phenix (Adams et al., 2011; Liebschner et al., 2019). The model was refined manually by Coot (Emsley et al., 2010). Structural similarity search was carried out with the DALI server (Holm and Laakso, 2016; Holm, 2020). All the figures of structures were generated using PyMOL software (DeLano, 2002; Schrödinger LLC, 2010)^3^.

### Sequence Alignment and 3D Structure Comparison

The amino acid sequence of E53 was analyzed by BLASTp against both the National Center for Biotechnology Information (NCBI) database^4^ and ESTHER database (Lenfant et al., 2012). Multiple sequence alignment was performed by ClustalX version 2.0 (Larkin et al., 2007) and ESPript version 3.0 (Robert and Gouet, 2014). The corresponding phylogenetic tree was constructed using the neighbor-joining method with MEGA version 7.0 (Kumar et al., 2015). The crystal structure data of family IV esterases were obtained from the RCSB protein data bank^5^. The structural comparison was conducted by PyMOL software (DeLano, 2002; Schrödinger LLC, 2010).

## Data Availability Statement

The datasets presented in this study can be found in online repositories. The names of the repository/repositories and accession number(s) can be found below: http://www.wwpdb.org/, 7W8N; http://www.wwpdb.org/, 7CI0; http://www.wwpdb.org/, 6KF5; and http://www.wwpdb.org/, 7CIH.

## Author Contributions

YD, LN, YG, X-CY, JL, and X-WX contributed to conception and study design. YD and Y-YH were responsible for X-ray diffraction. YD, YG, and YL were responsible for protein expression and enzymatic analysis and prepared all the figures, tables, and Supplementary Material. X-CY, Y-YH, and ZL contributed to crystal structure analysis. YD, LN, X-CY, JL, and X-WX were responsible for the interpretation of data. LN, X-CY, YL, JL, and X-WX were responsible for manuscript writing. All authors commented on previous versions of the manuscript. All authors read and approved the final manuscript.

## Conflict of Interest

LN was employed by the company CRELUX GmbH. The remaining authors declare that the research was conducted in the absence of any commercial or financial relationships that could be construed as a potential conflict of interest.

## Publisher’s Note

All claims expressed in this article are solely those of the authors and do not necessarily represent those of their affiliated organizations, or those of the publisher, the editors and the reviewers. Any product that may be evaluated in this article, or claim that may be made by its manufacturer, is not guaranteed or endorsed by the publisher.

## Footnotes

1
https://www.ncbi.nlm.nih.gov/nuccore/JMIW01000001.1?from=562979&to=563923&report=fasta

2
https://www.graphpad.com/quickcalcs/

3
http://pymol.sourceforge.net

4
https://blast.ncbi.nlm.nih.gov

5
https://www.rcsb.org

## Abbreviations

NP: Nitrophenyl
AAPB: Aerobic Anoxygenic Phototrophic Bacteria
PDB: Protein Data Bank
NCBI: National Center for Biotechnology Information.

## References

[B1] Adams P. Afonine P. Bunkóczi G. Chen V. Echols N. Headd J. (2011). The Phenix software for automated determination of macromolecular structures. *Methods* 55 94–106. 10.1016/j.ymeth.2011.07.005 21821126 PMC3193589

[B2] Angkawidjaja C. Koga Y. Takano K. Kanaya S. (2012). Structure and stability of a thermostable carboxylesterase from the thermoacidophilic archaeon *Sulfolobus tokodaii*. *FEBS J.* 279 3071–3084. 10.1111/j.1742-4658.2012.08687.x 22748144

[B3] Aouf C. Durand E. Lecomte J. Figueroa-Espinoza M.-C. Dubreucq E. Fulcrand H. (2014). The use of lipases as biocatalysts for the epoxidation of fatty acids and phenolic compounds. *Green Chem.* 16 1740–1754. 10.1039/c3gc42143k

[B4] Arpigny J. Jaeger K.-E. (1999). Bacterial lipolytic enzymes: classification and properties. *Biochem. J.* 343 177–183. 10.1042/0264-6021:3430177 10493927 PMC1220539

[B5] Castilla A. Panizza P. Rodriguez D. Bonino L. Diaz P. Irazoqui G. (2017). A novel thermophilic and halophilic esterase from *Janibacter* sp. R02, the first member of a new lipase family (Family XVII). *Enzyme Microb. Technol.* 98 86–95. 10.1016/j.enzmictec.2016.12.010 28110668

[B6] Cavazzini D. Grossi G. Levati E. Vallese F. Montanini B. Bolchi A. (2017). A family of archaea-like carboxylesterases preferentially expressed in the symbiotic phase of the mychorrizal fungus *Tuber melanosporum*. *Sci. Rep.* 7:7628. 10.1038/s41598-017-08007-9 28794466 PMC5550427

[B7] DeLano W. L. (2002). The PyMol molecular graphics system. *Proteins* 30 442–454.

[B8] Emsley P. Lohkamp B. Scott W. G. Cowtan K. D. (2010). Features and development of COOT. *Acta Crystallogr. D Biol. Crystallogr.* 66 486–501. 10.1107/S0907444910007493 20383002 PMC2852313

[B9] Ferrer M. Bargiela R. Martínez-Martínez M. Mir J. Koch R. Golyshina O. V. (2016). Biodiversity for biocatalysis: a review of the α/β-hydrolase fold superfamily of esterases-lipases discovered in metagenomes. *Biocatal. Biotransfor.* 33 235–249. 10.3109/10242422.2016.1151416

[B10] Fotheringham I. G. Grinter N. Pantaleone D. P. Senkpeil R. F. Taylor P. P. (1999). Engineering of a novel biochemical pathway for the biosynthesis of l-2-aminobutyric acid in *Escherichia coli* K12. *Bioorgan. Med. Chem.* 7 2209–2213. 10.1016/S0968-0896(99)00153-4 10579528

[B11] Gao H. Li C. Ramesh B. Liu Z. Hu N. Yong Q. (2018). A novel cold-adapted esterase from *Enterobacter cloacae*: characterization and improvement of its activity and thermostability *via* the site of Tyr193Cys. *Microb. Cell Fact.* 17:45. 10.1186/s12934-018-0885-z 29554914 PMC5858142

[B12] Garcia-Chaves C. Cottrell M. Kirchman D. Ruiz-González C. Giorgio P. (2016). Single-cell activity of freshwater aerobic anoxygenic phototrophic bacteria and their contribution to biomass production. *ISME J.* 10 1579–1588. 10.1038/ismej.2015.242 26771928 PMC4918449

[B13] Gasteiger E. Gattiker A. Hoogland C. Ivanyi I. Appel R. D. Bairoch A. (2003). ExPASy: the proteomics server for in-depth protein knowledge and analysis. *Nucleic Acids Res.* 31 3784–3788. 10.1093/nar/gkg563 12824418 PMC168970

[B14] Gonzalo G. D. Brieva R. Sánchez V. M. Bayod M. Gotor V. (2001). Enzymatic resolution of trans-4-(4’-fluorophenyl)-3-hydroxymethyl piperidines, key intermediates in the synthesis of -paroxetine. *J. Org. Chem.* 66 8947–8953. 10.1021/jo010809 11749627

[B15] Holm L. (2020). “Using dali for protein structure comparison,” in *Structural Bioinformatics*, ed. Gáspári Z. (Cham: Springer), 29–42. 10.1007/978-1-0716-0270-6_3 32006276

[B16] Holm L. Laakso L. (2016). Dali server update. *Nucleic Acids Res.* 44 gkw357. 10.1093/nar/gkw357 27131377 PMC4987910

[B17] Hotta Y. Ezaki S. Atomi H. Imanaka T. (2002). Extremely stable and versatile carboxylesterase from a hyperthermophilic archaeon. *Appl. Environ. Microbiol.* 68 3925–3931. 10.1128/AEM.68.8.3925-3931.2002 12147492 PMC124002

[B18] Houde A. Kademi A. Leblanc D. (2004). Lipases and their industrial applications: an overview. *Appl. Biochem. Biotechnol.* 118 155–170. 10.1385/ABAB:118:1-3:155 15304746

[B19] Huo Y.-Y. Rong Z. Jian S.-L. Xu C.-D. Li J. Xu X.-W. (2017). A novel halotolerant thermoalkaliphilic esterase from marine bacterium *Erythrobacter seohaensis* SW-135. *Front. Microbiol.* 8 2315–2326. 10.3389/fmicb.2017.02315 29213264 PMC5702849

[B20] Jeon J. Lee H. S. Kim J. Kim S.-J. Choi S. Kang S. (2011). Identification of a new subfamily of salt-tolerant esterases from a metagenomic library of tidal flat sediment. *Appl. Microbiol. Biotechnol.* 93 623–631. 10.1007/s00253-011-3433-x 21720822

[B21] Jose C. Toledo M. V. Briand L. E. (2016). Enzymatic kinetic resolution of racemic ibuprofen: past, present and future. *Crit. Rev. Biotechnol.* 36 891–903. 10.3109/07388551.2015.1057551 26121932

[B22] Kim Y. Park J. Kim M.-J. (2011). Dynamic kinetic resolution of amines and amino acids by enzyme-metal cocatalysis. *ChemCatChem* 3 271–277. 10.1002/cctc.201000330

[B23] Kobayashi R. Hirano N. Kanaya S. Haruki M. (2012). Enhancement of the enzymatic activity of *Escherichia coli* acetyl esterase by a double mutation obtained by random mutagenesis. *Biosci. Biotechnol. Biochem.* 76 2082–2088. 10.1271/bbb.120430 23132590

[B24] Kovalevskiy O. Nicholls R. A. Long F. Carlon A. Murshudov G. N. (2018). Overview of refinement procedures within REFMAC5: utilizing data from different sources. *Acta Crystallogr. D Struct. Biol.* 74(Pt 3) 215–227. 10.1107/S2059798318000979 29533229 PMC5947762

[B25] Kumar S. Stecher G. Tamura K. (2015). MEGA7: molecular evolutionary genetics analysis version 7.0 for bigger datasets. *Mol. Biol. Evol.* 33 1870–1874. 10.1093/molbev/msw054 PMC8210823 27004904

[B26] Larkin M. Blackshields G. Brown N. Chenna R. McGettigan P. McWilliam H. (2007). Clustal W and Clustal X version 2.0. *Bioinformatics* 23 2947–2948. 10.1093/bioinformatics/btm404 17846036

[B27] Lenfant N. Hotelier T. Velluet E. Bourne Y. Marchot P. Chatonnet A. (2012). ESTHER, the database of the α/β-hydrolase fold superfamily of proteins: tools to explore diversity of functions. *Nucleic Acids Res.* 41 423–429. 10.1093/nar/gks1154 23193256 PMC3531081

[B28] Li P.-Y. Chen X.-L. Ji P. Li C.-Y. Wang P. Zhang Y. (2015). Inter-domain hydrophobic interactions modulate the thermostability of microbial esterases from the hormone-sensitive lipase family. *J. Biol. Chem.* 290 11188–11198. 10.1074/jbc.M115.646182 25771540 PMC4409275

[B29] Li P.-Y. Ji P. Li C.-Y. Zhang Y. Wang G.-L. Zhang X.-Y. (2014). Structural basis for dimerization and catalysis of a novel esterase from the GTSAG motif subfamily of bacterial hormone-sensitive lipase (HSL) family. *J. Biol. Chem.* 289 19031–19041. 10.1074/jbc.M114.574913 24867954 PMC4081941

[B30] Liebschner D. Afonine P. V. Baker M. L. Bunkoczi G. Chen V. B. Croll T. I. (2019). Macromolecular structure determination using X-rays, neutrons and electrons: recent developments in Phenix. *Acta Crystallogr. D Struct. Biol.* 75(Pt 10) 861–877. 10.1107/S2059798319011471 31588918 PMC6778852

[B31] Manco G. Giosuè E. D’Auria S. Herman P. Carrea G. Rossi M. (2000). Cloning, overexpression, and properties of a new thermophilic and thermostable esterase with sequence similarity to hormone-sensitive lipase subfamily from the archaeon *Archaeoglobus fulgidus*. *Arch. Biochem. Biophys.* 373 182–192. 10.1006/abbi.1999.1497 10620337

[B32] Mandrich L. Merone L. Pezzullo M. Cipolla L. Nicotra F. Rossi M. (2005). Role of the N terminus in enzyme activity, stability and specificity in thermophilic esterases belonging to the HSL family. *J. Mol. Biol.* 345 501–512. 10.1016/j.jmb.2004.10.035 15581894

[B33] Martinez M. Alcaide M. Tchigvintsev A. Reva O. Polaina J. Bargiela R. (2013). Biochemical diversity of carboxyl esterases and lipases from lake Arreo (Spain): a metagenomic approach. *Appl. Environ. Microbiol.* 79 3553–3562. 10.1128/AEM.00240-13 23542620 PMC3675924

[B34] Martinez M. Coscolín C. Santiago G. Chow J. Stogios P. Bargiela R. (2017). Determinants and prediction of esterase substrate promiscuity patterns. *ACS Chem. Biol.* 13 225–234. 10.1021/acschembio.7b00996 29182315

[B35] McCoy A. Grosse-Kunstleve R. Adams P. Winn M. Storoni L. Read R. (2007). PHASER crystallographic software. *J. Appl. Crystallogr.* 40 658–674. 10.1107/S0021889807021206 19461840 PMC2483472

[B36] Minor W. Cymborowski M. Otwinowski Z. Chruszcz M. (2006). HKL-3000: the integration of data reduction and structure solution – From diffraction images to an initial model in minutes. *Acta Crystallogr. D Biol. Crystallogr.* 62 859–866. 10.1107/S0907444906019949 16855301

[B37] Monti D. Gazák R. Marhol P. Biedermann D. Purchartová K. Fedrigo M. (2010). Enzymatic Kinetic resolution of silybin diastereoisomers. *J. Nat. Prod.* 73 613–619. 20297826 10.1021/np900758d

[B38] Moreth K. Riester D. Hildmann C. Hempel R. Wegener D. Schober A. (2007). An active site tyrosine residue is essential for amidohydrolase but not for esterase activity of a class 2 histone deacetylase-like bacterial enzyme. *Biochem. J.* 401 659–665. 10.1042/BJ20061239 17037985 PMC1770855

[B39] Murshudov G. Skubak P. Lebedev A. Pannu N. Steiner R. A. Nicholls R. (2011). REFMAC5 For the refinement of macromolecular crystal structures. *Acta Crystallogr. D Biol. Crystallogr.* 67 355–367. 10.1107/S0907444911001314 21460454 PMC3069751

[B40] Nardini M. Dijkstra B. (1999). Alpha/beta hydrolase fold enzymes: the family keeps growing. *Curr. Opin. Struct. Biol.* 9 732–737. 10.1016/S0959-440X(99)00037-8 10607665

[B41] Oh J. Hwang I. Rhee S. (2016). Structural insights into an oxalate-producing serine hydrolase with an unusual oxyanion hole and additional lyase activity. *J. Biol. Chem.* 291 15185–15195. 10.1074/jbc.M116.727180 27226606 PMC4946933

[B42] Ohara K. Unno H. Oshima Y. Hosoya M. Fujino N. Hirooka K. (2014). Structural insights into the low-pH adaptation of a unique carboxylesterase from *Ferroplasma*: altering the pH optima of two carboxylesterases. *J. Biol. Chem.* 289 24499–24510. 10.1074/jbc.M113.521856 25043762 PMC4148875

[B43] Palm G. Fernández-Álvaro E. Bogdanović X. Bartsch S. Sczodrok J. Singh R. (2011). The crystal structure of an esterase from the hyperthermophilic microorganism *Pyrobaculum calidifontis* VA1 explains its enantioselectivity. *Appl. Microbiol. Biotechnol.* 91 1061–1072. 10.1007/s00253-011-3337-9 21614503

[B44] Park J. M. Kang C. H. Won S. M. Oh K. H. Yoon J. H. (2019). Characterization of a novel moderately thermophilic solvent-tolerant esterase isolated from a compost metagenome library. *Front. Microbiol.* 10:3069. 10.3389/fmicb.2019.03069 32038535 PMC6993047

[B45] Pereira M. Maester T. Mercaldi G. Lemos E. Hyvönen M. Balan A. (2017). From a metagenomic source to a high-resolution structure of a novel alkaline esterase. *Appl. Microbiol. Biotechnol.* 101 4935–4949. 10.1007/s00253-017-8226-4 28331945

[B46] Petrovskaya L. Novototskaya-Vlasova K. Spirina E. Durdenko E. Lomakina G. Zavialova M. (2016). Expression and characterization of a new esterase with GCSAG motif from a permafrost metagenomic library. *FEMS Microbiol. Ecol.* 92:fiw046. 10.1093/femsec/fiw046 26929439

[B47] Robert X. Gouet P. (2014). Deciphering key features in protein structures with the new ENDscript Server. *Nucleic Acids Res.* 42 320–324. 10.1093/nar/gku316 24753421 PMC4086106

[B48] Samoylova Y. V. Sorokina K. N. Romanenko M. V. Parmon V. N. (2018). Cloning, expression and characterization of the esterase estUT1 from *Ureibacillus thermosphaericus* which belongs to a new lipase family XVIII. *Extremophiles* 22 271–285. 10.1007/s00792-018-0996-9 29330648

[B49] Santiago G. Martinez M. Alonso Rubido S. Bargiela R. Coscolín C. Golyshin P. (2018). Rational engineering of multiple active sites in an ester hydrolase. *Biochemistry* 57 2245–2255. 10.1021/acs.biochem.8b00274 29600855

[B50] Schrödinger LLC (2010). *The PyMOL Molecular Graphics System, Version 1.3r1.* New York, NY: Schrödinger, LLC.

[B51] Secundo F. Carrea G. Soregaroli C. Varinelli D. Morrone R. (2001). Activity of different *Candida antartica* lipase B formulations in organic solvents. *Biotechnol. Bioeng.* 73 157–163. 10.1002/bit.1047 11255163

[B52] Shiba T. Simidu U. (1982). *Erythrobacter longus* gen. nov., sp. nov., an aerobic bacterium which contains bacteriochlorophyll *a*. Int. J. Syst. Bacteriol. 32 211–217.

[B53] Stergiou P.-Y. Foukis A. Filippou M. Koukouritaki M. Parapouli M. Theodorou L. (2013). Advances in lipase-catalyzed esterification reactions. *Biotechnol. Adv.* 31 1846–1859. 10.1016/j.biotechadv.2013.08.006 23954307

[B54] Wang M. Ai L. Zhang M. Wang F. Wang C. (2020). Characterization of a novel halotolerant esterase from *Chromohalobacter canadensis* isolated from salt well mine. *3 Biotech* 10 430. 10.1007/s13205-020-02420-0 32983823 PMC7490289

[B55] Winn M. Ballard C. Cowtan K. Dodson E. Emsley P. Evans P. (2011). Overview of the CCP4 suite and current development. *Acta Crystallogr. D Biol. Crystallogr.* 67 235–242. 10.1107/S0907444910045749 21460441 PMC3069738

[B56] Xu L. Wu Y.-H. Cheng H. Sun C. Han B.-N. Xu X.-W. (2018). Complete genome sequence of *Erythrobacter seohaensis* SW-135T sheds light on the ecological role of the genus *Erythrobacter* for phosphorus cycle in the marine environment. *Mar. Genomics* 40 21–24. 10.1016/j.margen.2018.03.001 32420878

[B57] Yang S. Qin Z. Duan X. Yan Q. Jiang Z. (2015). Structural insights into the substrate specificity of two esterases from the thermophilic *Rhizomucor miehei*. *J. Lipid Res.* 56 1616–1624. 10.1194/jlr.M060673 26108223 PMC4514002

[B58] Yesiloglu Y. Kilic I. (2004). Lipase-catalyzed esterification of glycerol and oleic acid. *J. Am. Oil Chem. Soc.* 81 281–284. 10.1007/s11746-004-0896-5

