# Supplementary material for: Mechanism and Structural Insights Into a Novel Esterase, E53, Isolated From Erythrobacter longus
Source: Front Microbiol. 2022 Jan 5;12:798194. doi: 10.3389/fmicb.2021.798194 (PMC8767022; doi:10.3389/fmicb.2021.798194)
Supplement: Supplementary file 1 [file Data_Sheet_1.docx]

Supplementary Material

## Supplementary Figures


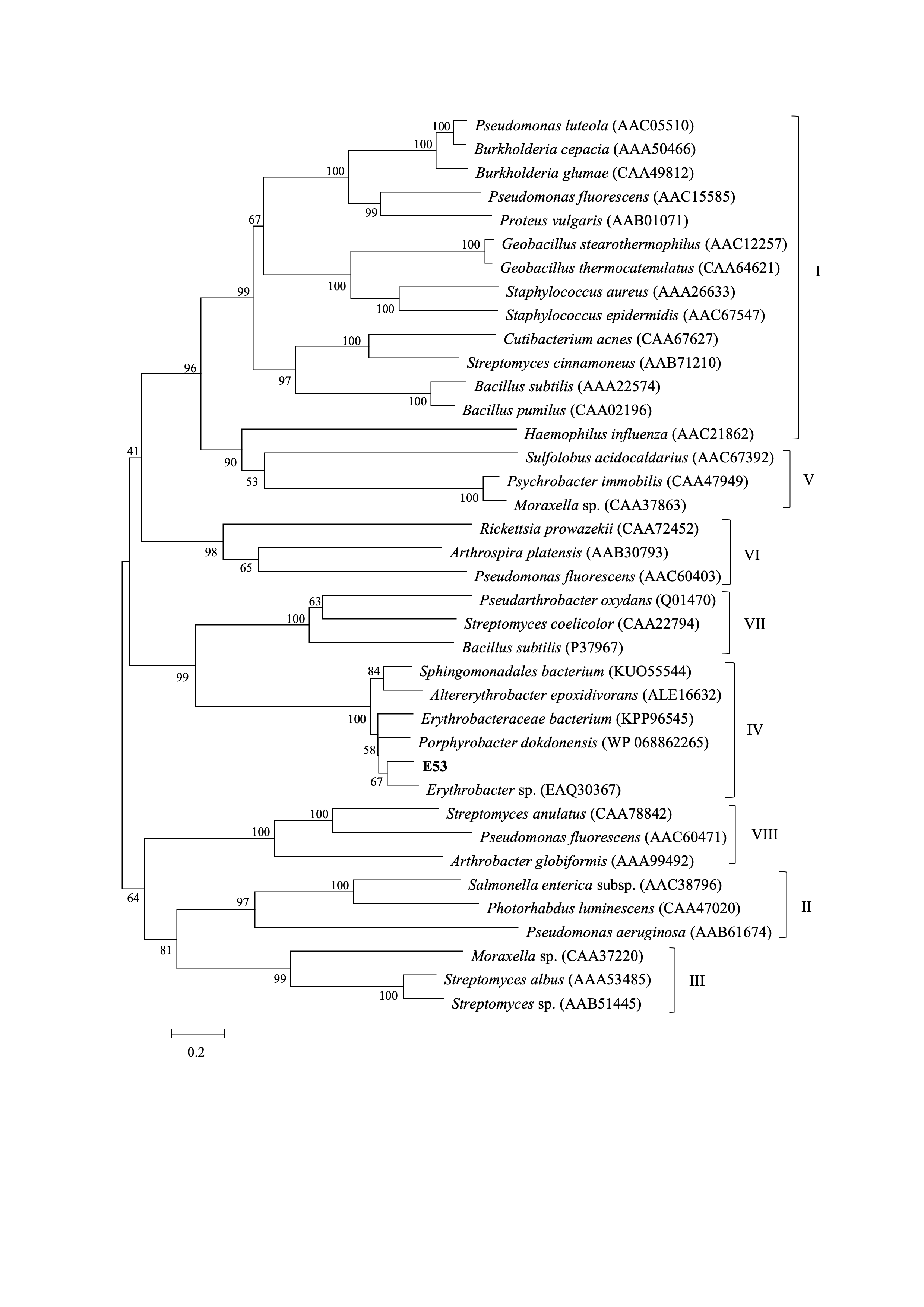


**Figure S1.** Phylogenetic tree based on amino acid sequences of E53 and its homologous proteins. Sequence alignment was performed using ClustalX software. The phylogenetic tree was constructed by the MEGA software. Bootstrap values were based on 1000 replicates and the values higher than 50 % were shown in the tree. The scale bar measured the number of amino acid substitutions per site.


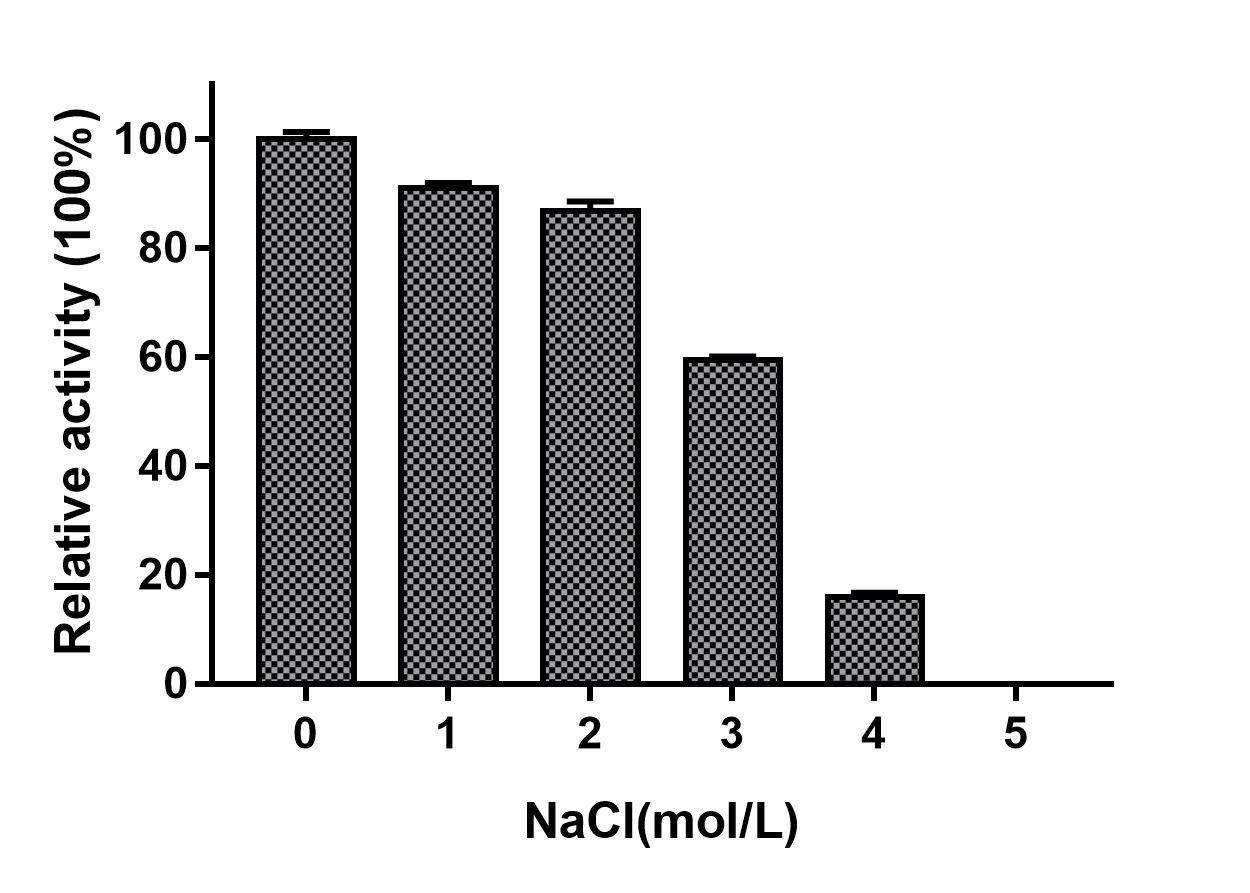


**Figure S2.** The catalytic activity of wild type (WT) E53 under different NaCl concentrations. The reaction without NaCl was used as a control.


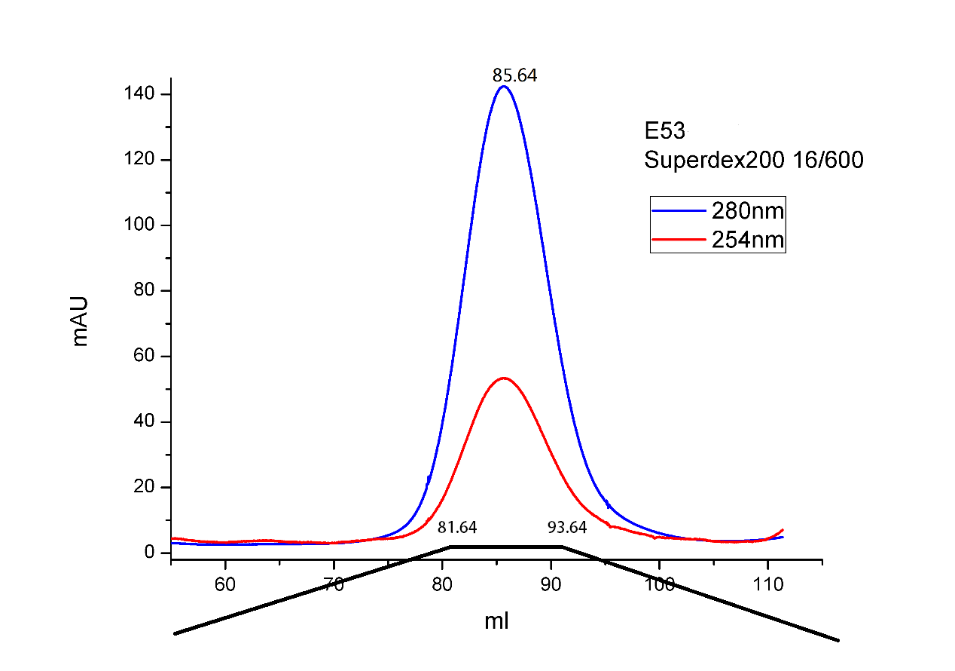

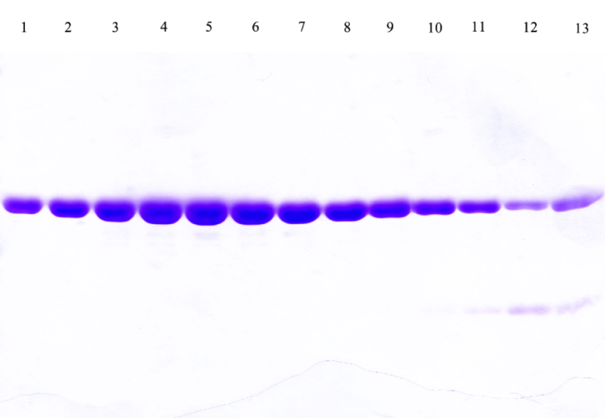


**Figure S3.** Molecular sieve UV absorption peak of WT E53. The sample was run on a Superdex 200 16/600 GL column and was eluted at 85.64 ml.

**
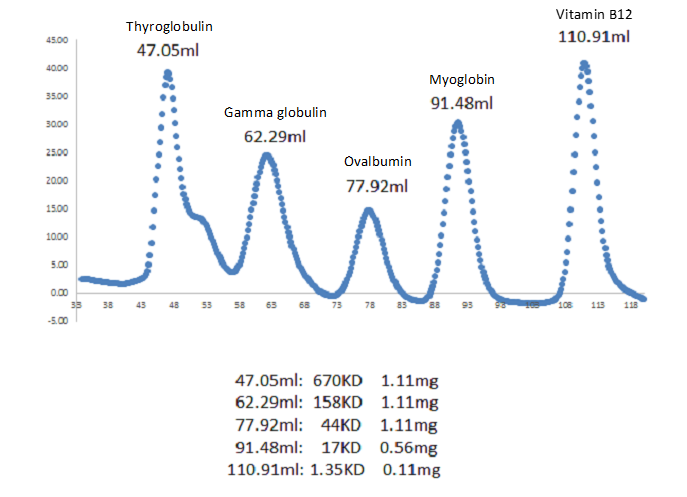
**

B

A

**
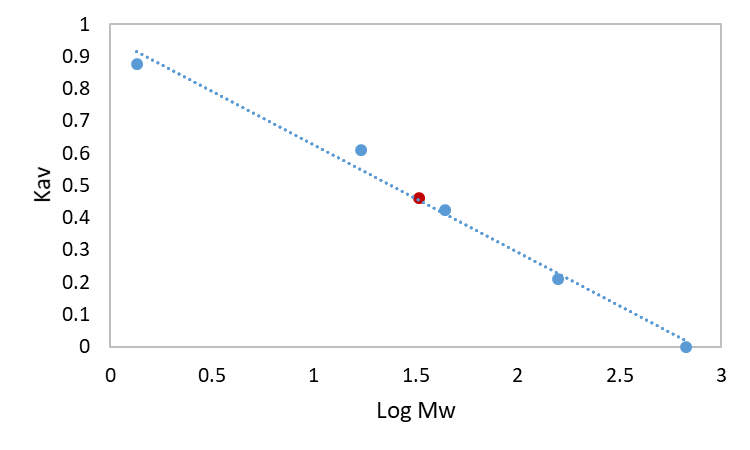
**

***E53***

***32.4 kDa***

Thyroglobulin

670 kDa

Gamma globulin

158 kDa

Ovalbumin

44 kDa

Myoglobin

17 kDa

Vitamin B12

1.35 kDa

**Figure S4.** Determination of Mw of E53. **(A).** The Superdex 200 16/600 GL standard elution curve. Protein size markers and corresponding elution volume are indicated in the figure. **(B)**. Calibration curve of the The Superdex 200 16/600 GL column. Protein markers are labelled in blue dots, E53 is in red dot. Protein name and corresponding molecular weight is labelled beside the dot. The fitting has an R^2^ of 0.987.


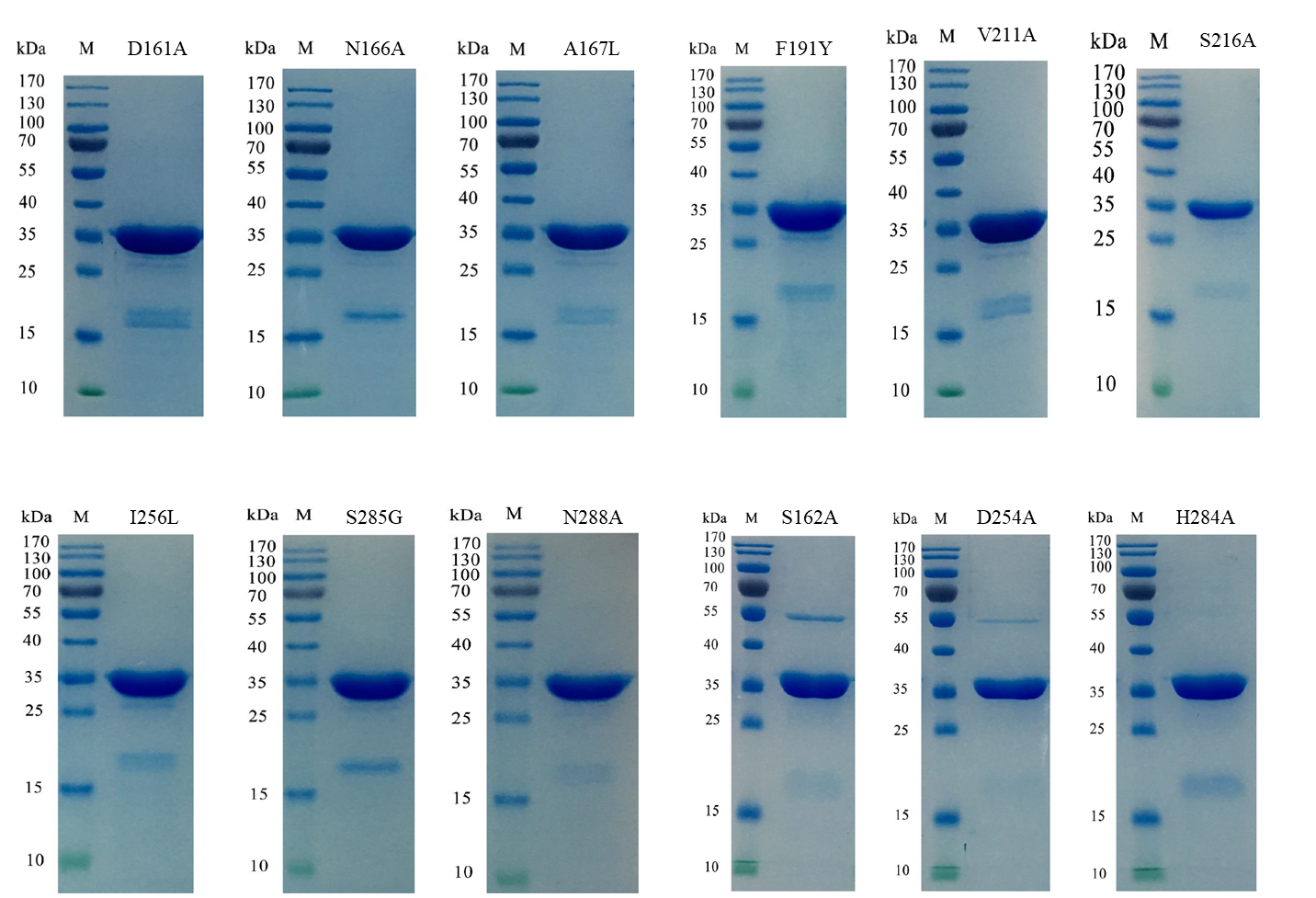


**Figure S5**. SDS-PAGE of all variants used in this study. The marker and variant are labelled in the figures.


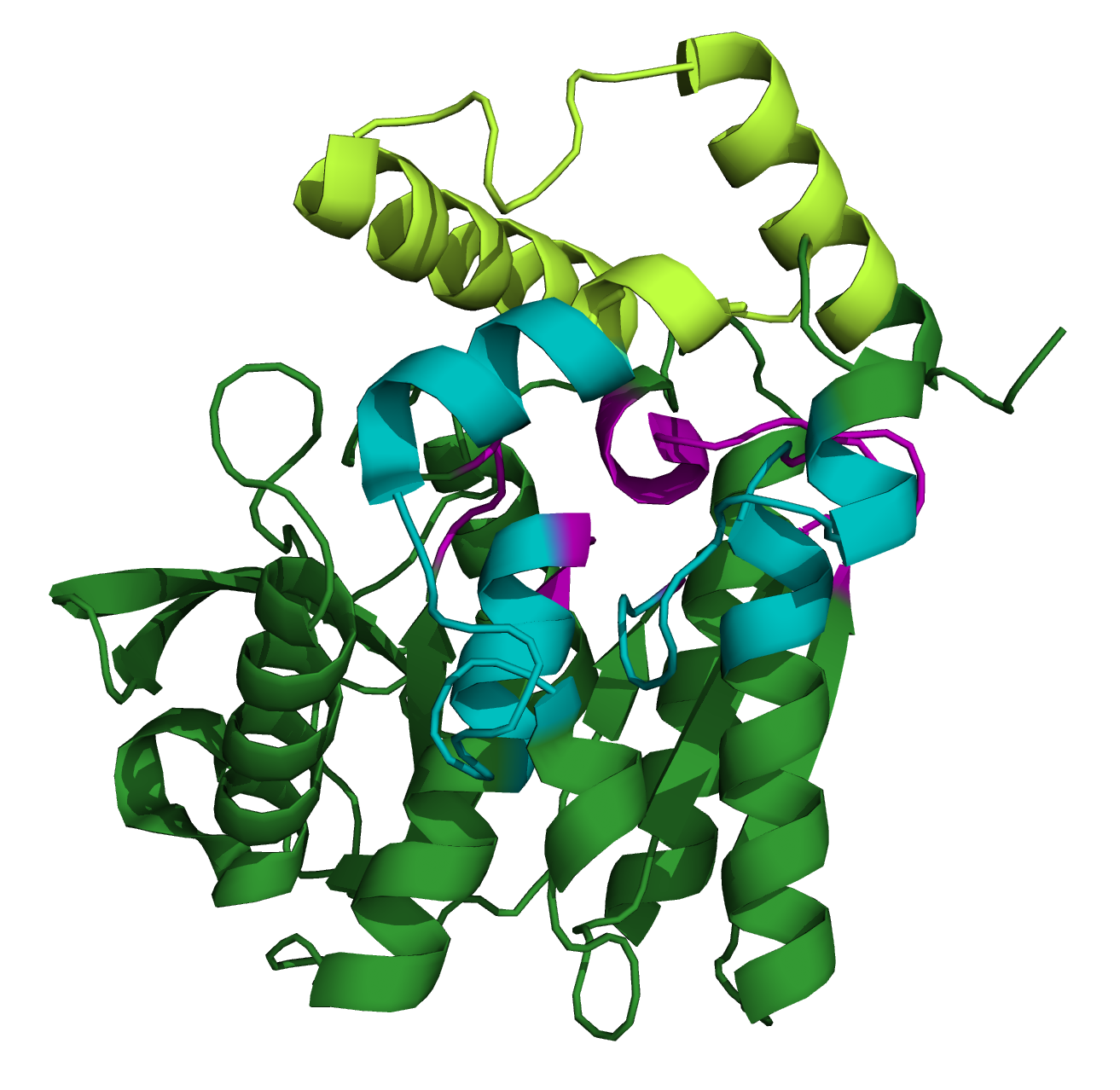


**Figure S6.** Visualization of R1, R2 and R3 region. The backbone of E53 is shown in green. R1 region is in magenta, R2 region is in yellow and R3 region is in cyan. The amino acid residues involved in each region are as following: R1: HGGG (88-91), GDS (160-162), F191, ASLD (251-254) and MEGVTHSFTN (279-288); R2: KAFLEAIAAMAGPTLAEMTLEEARASYVALHGMAD (11-47), GFV (209-211) and TKAS (213-216); R3: AGGNATI (163-169), PLASDAVGSASL (192-203), IEFFDTAYKADRADPRGF (217-234) and PIRDS (255-259)


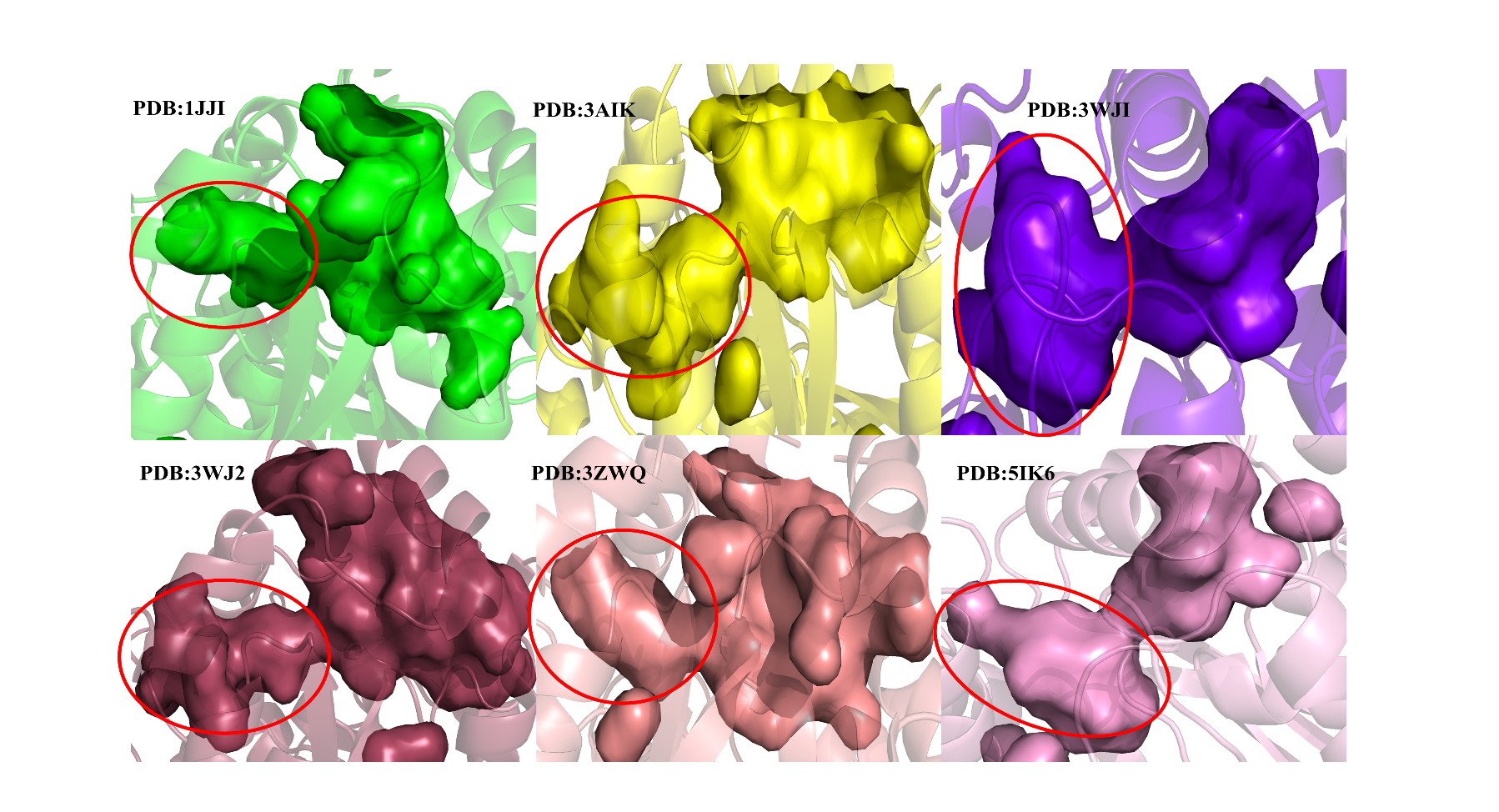

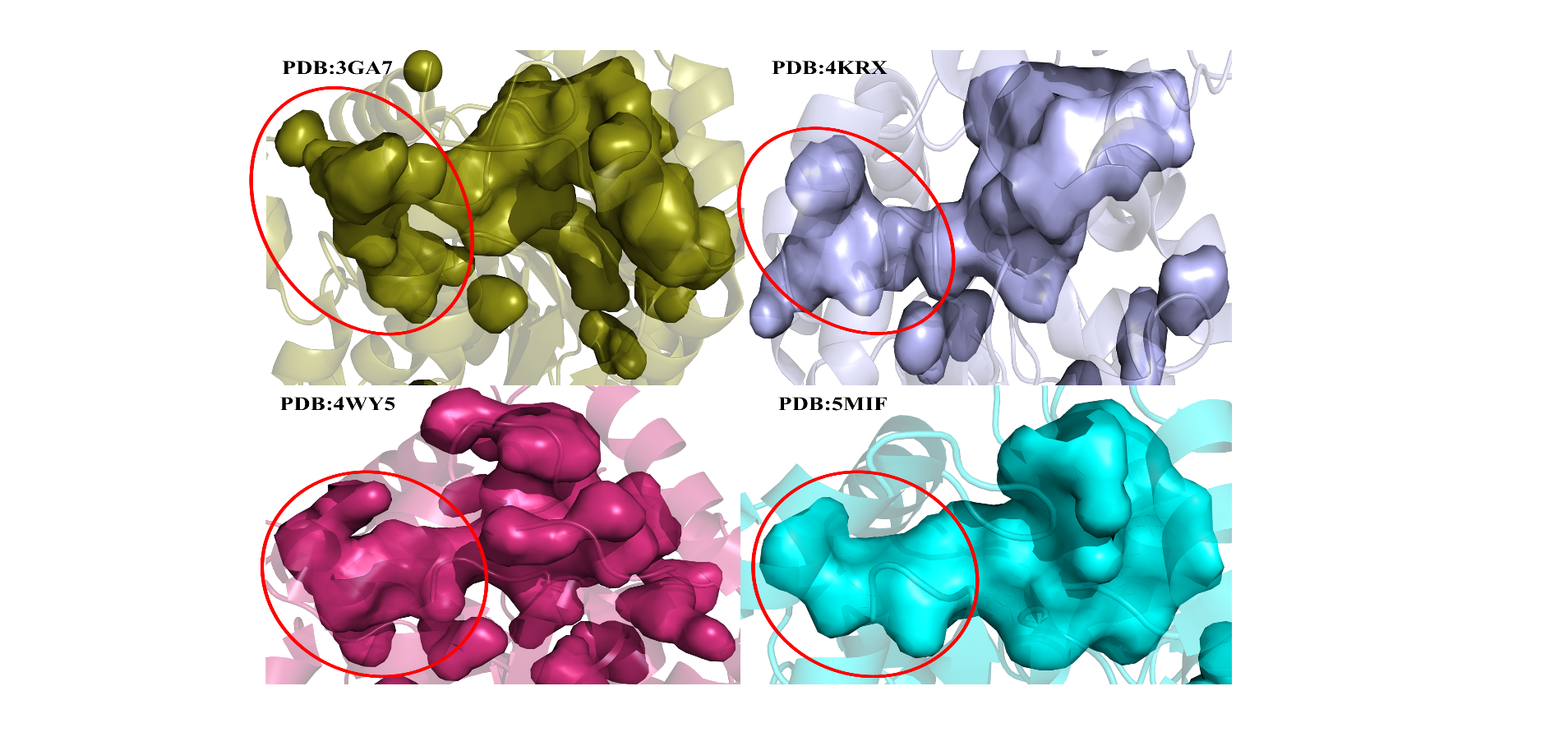


**Figure S7.** The microbial family IV esterases that contain the blind end area at the catalytic pocket were shown in cartoon. The blind end area was depicted by a red circle.


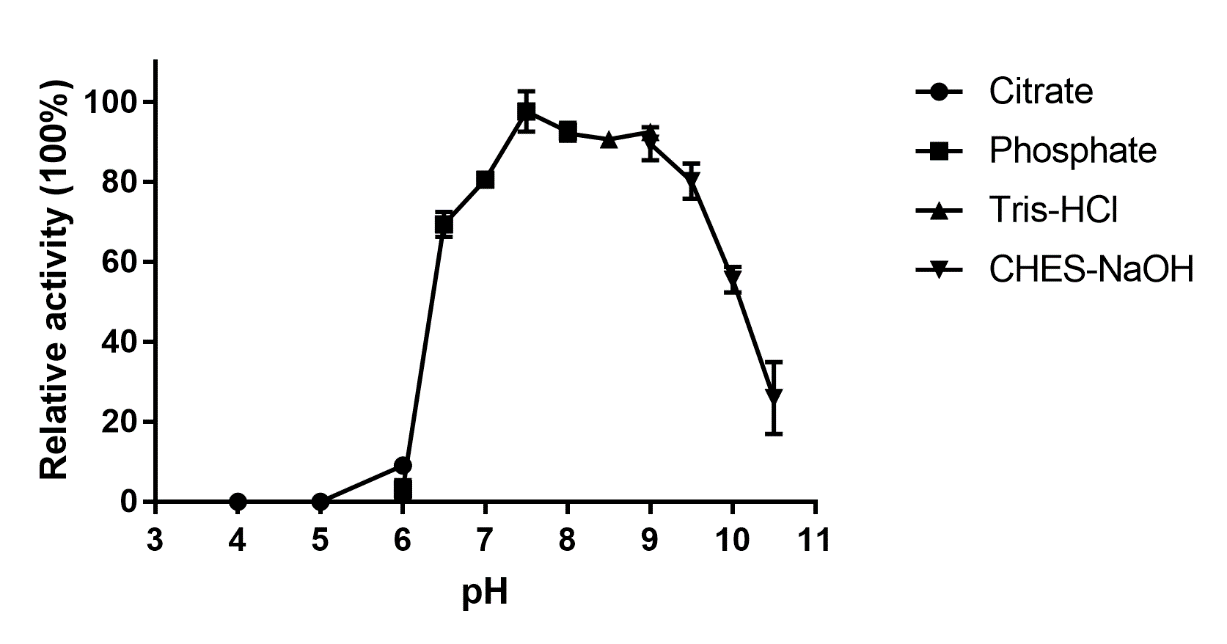


**Figure S8.** Effect of pH value on the activity of E53 N166A variant. The data are shown as mean ±SD (n=3). For some points, the error bars would be shorter than the height of the symbol.

**
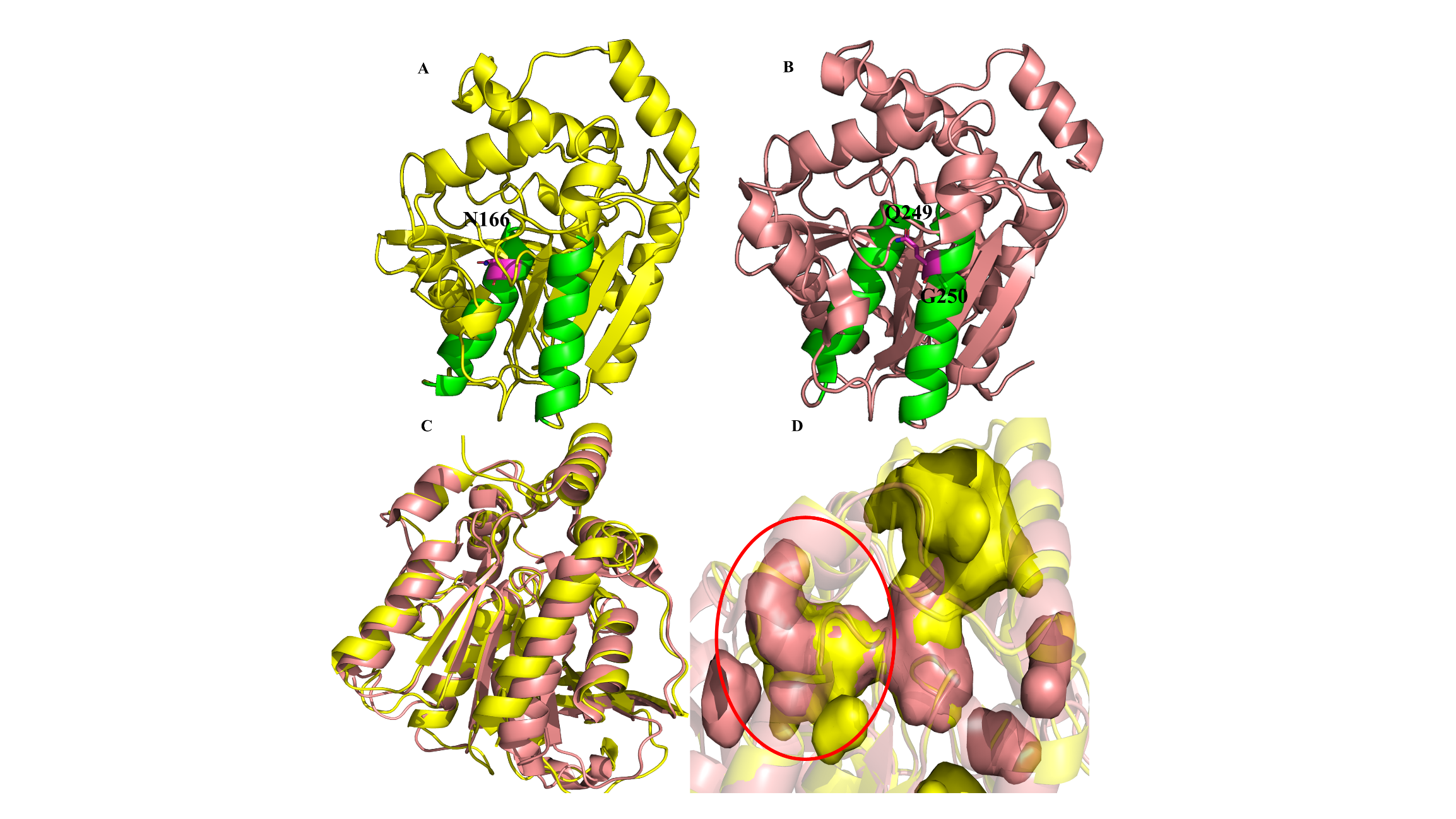
**

**Figure S9**. Structural comparison between E53 and SshEst. A, E53 was shown in cartoon with two α helices (α5 and α8) labelled with green color. The N166 located at the α5 helix was depicted with purple color. B, SshEst was shown in cartoon with two α helices (α5 and α8) labelled with green color. The Q249 and G250 resides located at the α8 helix were depicted with purple color. C, Structure superimposition of E53 (yellow) and SshEst (salmon). D, The catalytic pocket surfaces of E53 (yellow) and SshEst (salmon) were shown. Both enzymes contain the blind end area.


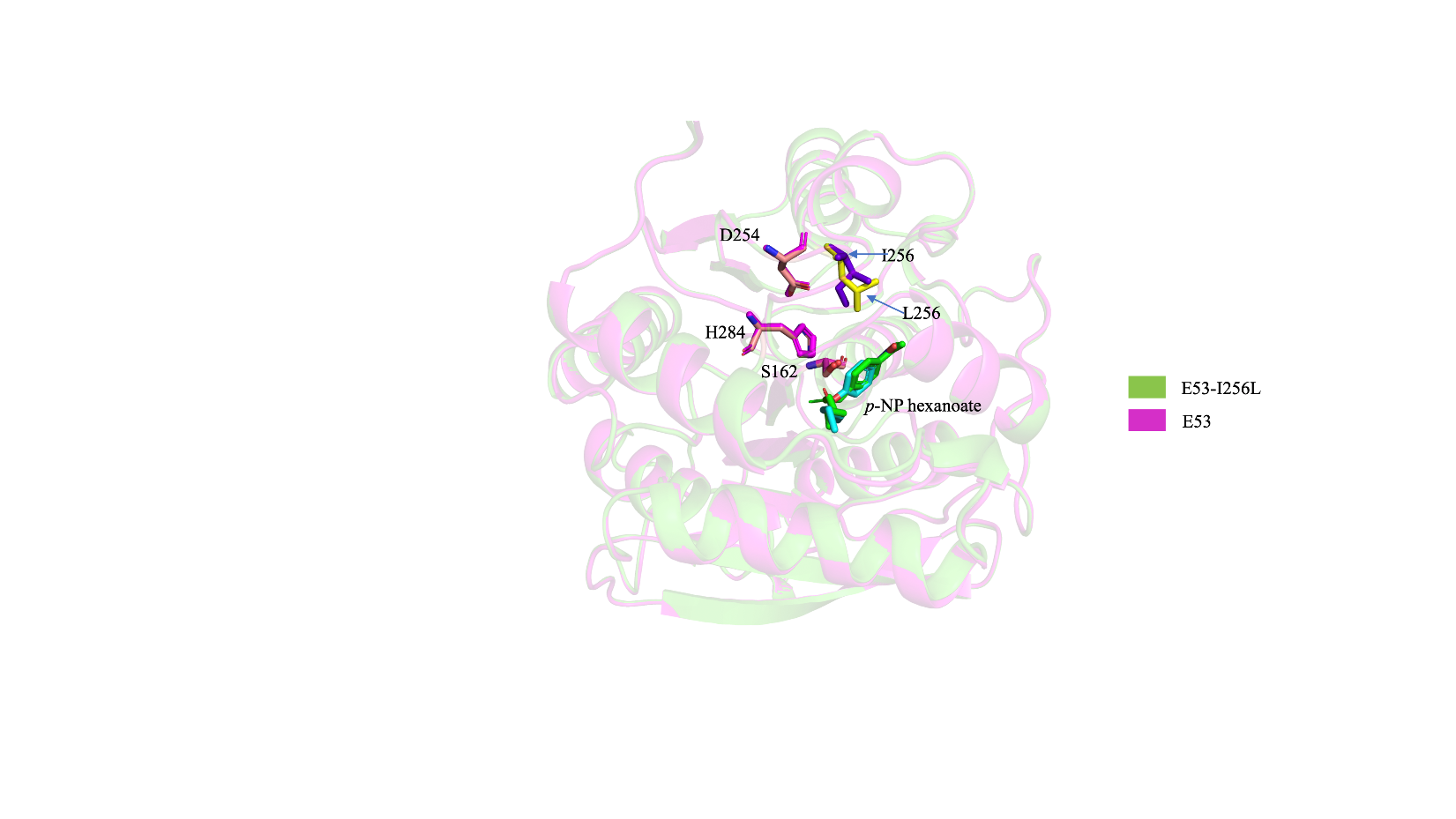


**Figure S10**. Structural comparison for E53 and its mutant I256L. The catalytic traid S162, D254 and H284 was shown in stick models

**
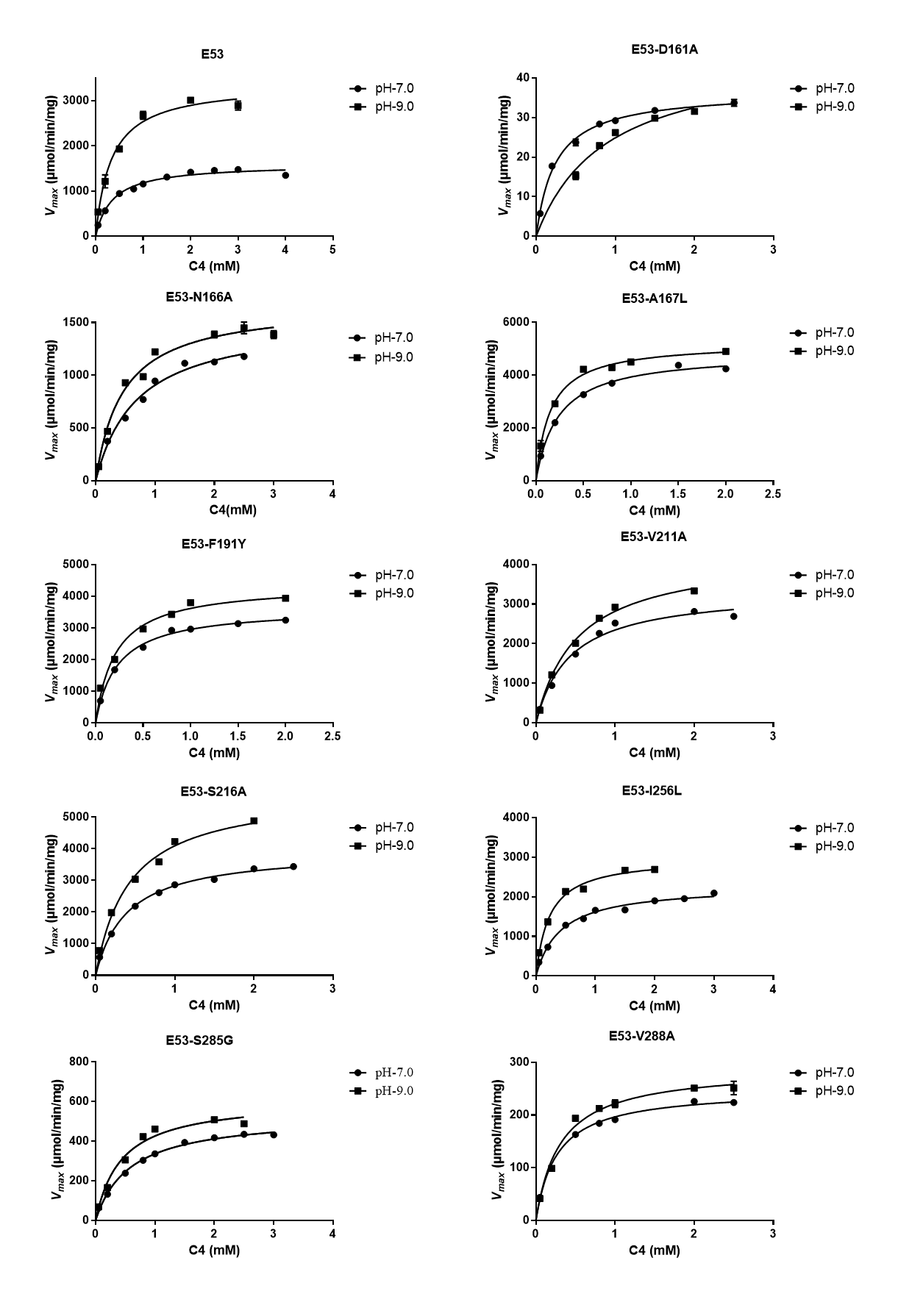
**

**Figure S11**. Kinetic curves of E53 WT and its variants in neutral and alkaline environments

**Supplementary Tables**

**Table S1.** The amino acid sequence alignment and the homology analysis of E53.

| **Name** | **PDB accession number** | **Identity** | **Origin** | **Microorganism** |
| --- | --- | --- | --- | --- |
| Est8 | 4YPV | 125/291 (43%) | Metagenome | - |
| Sto-Est | 3AIK | 99/286 (35%) | *Sulfolobus tokodaii* | Archaeon |
| AFEST | 1JJI | 94/279 (34%) | *Archaeoglobus fulgidus* | Archaeon |
| PestE | 2YH2 | 107/314 (34%) | *Pyrobaculum calidifontis* | Archaeon |
| Esta | 5LK6 | 97/267 (36%) | *Sulfolobus islandicus* REY15A | Archaeon |
| SshEstI | 3WJ1 | 98/286 (34%) | *Sulfolobus shibatae* DSM5389 | Archaeon |
| Lae6 | 5JD4 | 104/254 (41%) | Metagenome | - |
| ThaEst2349 | 4V2I | 99/303 (33%) | *Thalassospira* sp. GB04J01 | Bacterium |
| Este1 | 2C7B | 105/292 (36%) | Metagenome | - |
| Est2 | 1EVQ | 96/308 (31%) | *Alicyclobacillus acidocaldarius* | Bacterium |
| rPPE | 4OB8 | 96/319 (30%) | *Pseudomonas putida* ECU1011 | Bacterium |
| EstFa_R | 3WJ2 | 75/240 (31%) | *Ferroplasma acidiphilum* | Archaeon |

**Table S2.** The microbial family IV esterases with crystal structures in the ESTHER database.

| **PDB ID** | **Resources** | **Organisms** | **R3-similiar region** |
| --- | --- | --- | --- |
| 1JJI | *Archaeoglobus fulgidus* | Archaeon | X |
| 3AIK | *Sulfolobus tokodaii* | Archaeon | X |
| 3WJ1 | *Sulfolobus shibatae* | Archaeon | X |
| 3WJ2 | *Ferroplasma acidiphilum* | Archaeon | X |
| 3ZWQ | *Pyrobaculum calidifontis* | Archaeon | X |
| 5L2P | *Saccharolobus solfataricus* | Archaeon |  |
| 5LK6 | *Sulfolobus islandicus* | Archaeon | X |
| 1EVQ | *Alicyclobacillus acidocaldarius* | Bacterium |  |
| 1JKM | *Bacillus subtilis* | Bacterium |  |
| 1LZL | *Rhodococcus* sp. | Bacterium |  |
| 2PBL | *Silicibacter* sp. | Bacterium |  |
| 2QRU | *Enterococcus faecalis* | Bacterium |  |
| 3D7R | *Staphylococcus aureus* | Bacterium |  |
| 3GA7 | *Salmonella typhimurium.* | Bacterium | X |
| 3H04 | *Staphylococcus aureus* | Bacterium |  |
| 3HXK | *Lactococcus lactis* | Bacterium |  |
| 3QH4 | *Mycobacterium marinum* | Bacterium |  |
| 4C89 | *Lactobacillus plantarum* | Bacterium | X |
| 4KRX | *Escherichia coli* | Bacterium | X |
| 4N5H | *Lactobacillus rhamnosis* | Bacterium |  |
| 4OB8 | *Pseudomonas putida* | Bacterium |  |
| 4V2I | *Thalassospira* sp. | Bacterium |  |
| 5AO9 | *Thermogutta terrifontis* | Bacterium |  |
| 4WY5 | *Rhizomucor miehei* | Fungus | X |
| 4WY8 | *Rhizomucor miehei* | Fungus |  |
| 5MIF | *Tuber melanosporum* | Fungus | X |
| 2C7B | Metagenomic Library | - |  |
| 3DNM | Metagenome Library | - |  |
| 3FAK | Metagenome Library | - |  |
| 4J7A | Metagenome Library | - |  |
| 4Q3K | Metagenome Library | - |  |
| 4YPV | Metagenome Library | - |  |
| 4ZRS | Metagenome Library | - |  |
| 5GMS | Metagenome Library | - |  |
| 5HC4 | Metagenome Library | - |  |
| 5JD4 | Metagenome Library | - | X |
| 5JD5 | Metagenome Library | - |  |
| 6AAE | Metagenome Library | - |  |

**Table S3.** Kinetic data of E53 and its mutants.

| E53 and mutants | *V*_max_ (umol/min/mg) | | *K_m_* (mM) | |
| --- | --- | --- | --- | --- |
|  | pH7.0 | pH9.0 | pH7.0 | pH9.0 |
| E53 | 1596±27 | 3347±88 | 0.35±0.03 | 0.33±0.03 |
| E53-D161A | 37±1* | 47±2* | 0.24±0.01* | 0.88±0.11* |
| E53-N166A | 1540±46 | 1678±40* | 0.70±0.06* | 0.46±0.04* |
| E53-A167L | 4860±69* | 5238±73* | 0.24±0.01* | 0.15±0.01* |
| E53-F191Y | 3639±41* | 4381±103* | 0.23±0.01* | 0.21±0.02* |
| E53-V211A | 3359±93* | 4280±93* | 0.43±0.04 | 0.52±0.03* |
| E53-S216A | 3956±48* | 5832±162* | 0.40±0.02 | 0.42±0.03* |
| E53-I256L | 2285±44* | 2999±52* | 0.41±0.03 | 0.23±0.02* |
| E53-S285G | 531±8* | 613±20* | 0.58±0.03* | 0.44±0.05* |
| E53-N288A | 249±3* | 290±7* | 0.27±0.01* | 0.31±0.03 |

The data are shown as mean ±SD (n=3).

*: Representing a significant difference from the WT E53 (*t* test, p < 0.05).**Table S4.** Primers for *e53* gene amplification and mutants.

| **Primers** | **Sequences** |
| --- | --- |
| E53-F | TCGCGGATCCATGACCGACACACCCTTC |
| E53-R | TCCGCTCGAGTCAGGCAGTCCCCAACATC |
| D161A-F | GGGCGTCATTCCCATCGGCGCCAGCGCAGGCG |
| D161A-R | GCGTGCCGCAGCCCGCAGTAAGGGTAGCCGCG |
| N166A-F | TCGGCGACAGCGCAGGCGGAGCTGCGACCATCG |
| N166A-R | GCAGTAAGGGTAGCCGCTGTCGCGTCCGCCTCG |
| A167L-F | AGCGCAGGCGGAAATCTGACCATCGTGGTGAGC |
| A167L-R | GCTCACCACGATGGTCAGATTTCCGCCTGCGCT |
| F191Y-F | CTGCAAGTCCCGATCTACCCGCTCGCAAGCGAT |
| F191Y-R | ATCGCTTGCGAGCGGGTAGATCGGGACTTGCAG |
| V211A-F | AGCCTTTGCCGAAGGCTTTGCCCTTACCAAAGC |
| V211A-R | CCGGTCAGAACTTCGGAAACGGCTTCCGAAACG |
| S216A-F | GCTTTGTCCTTACCAAAGCGGCGATCGAATTTTTTGA |
| S216A-R | TTCGGAAACGGCTTCCGAAACAGGAATGGTTTCGCC |
| D254A-F | GCCACCGCCAGCCTTGCTCCGATCCGCGATTCC |
| D254A-R | GGAATCGCGGATCGGAGCAAGGCTGGCGGTGGC |
| I256L-F | GCCAGCCTTGATCCGCTCCGCGATTCCGGGCGC |
| I256L-R | GCGCCCGGAATCGCGGAGCGGATCAAGGCTGGC |
| S285G-F | GAAGGGGTCACCCATGGTTTCACCAATATTCGC |
| S285G-R | GCGAATATTGGTGAAACCATGGGTGACCCCTTC |
| N288A-F | GGGTCACCCATTCTTTCACCGCTATTCGCGCCGC |
| N288A-R | AGCTTTACCTTCCCCAGTGGGTAAGAAAGTGGCG |
